# A nitrogen fixing symbiosis-specific pathway required for legume flowering

**DOI:** 10.1126/sciadv.ade1150

**Published:** 2023-01-13

**Authors:** Jinxia Yun, Can Wang, Fengrong Zhang, Li Chen, Zhengxi Sun, Yupeng Cai, Yuanqing Luo, Junwen Liao, Yongliang Wang, Yanyan Cha, Xuehai Zhang, Ya Ren, Jun Wu, Paul M. Hasegawa, Changfu Tian, Huanan Su, Brett J. Ferguson, Peter M. Gresshoff, Wensheng Hou, Tianfu Han, Xia Li

**Affiliations:** ^1^ National Key Laboratory of Crop Genetic Improvement, Hubei Hongshan Laboratory, College of Plant Science and Technology, Huazhong Agricultural University, Wuhan, China.; ^2^ National Center for Transgenic Research in Plants, Institute of Crop Sciences, Chinese Academy of Agricultural Sciences, Beijing, China.; ^3^ Ministry of Agriculture Key Laboratory of Soybean Biology (Beijing), Institute of Crop Sciences, Chinese Academy of Agricultural Sciences, Beijing, China.; ^4^ Department of Horticulture and Landscape Architecture, Purdue University, West Lafayette, IN 47907, USA.; ^5^ State Key Laboratory of Agrobiotechnology, Key Laboratory of Soil Microbiology, and Rhizobium Research Center, and College of Biological Sciences, China Agricultural University, Beijing, China.; ^6^ School of Agriculture and Food Sciences, The University of Queensland, St Lucia, Brisbane, QLD 4072, Australia.; ^7^ Guangdong Laboratory for Lingnan Modern Agriculture, Guangdong, China.

## Abstract

Symbiotic nitrogen fixation boosts legume growth and production in nitrogen-poor soils. It has long been assumed that fixed nitrogen increases reproductive success, but until now, the regulatory mechanism was unknown. Here, we report a symbiotic flowering pathway that couples symbiotic and nutrient signals to the flowering induction pathway in legumes. We show that the symbiotic microRNA–microRNA172c (miR172c) and fixed nitrogen systemically and synergistically convey symbiotic and nutritional cues from roots to leaves to promote soybean (*Glycine max*) flowering. The combinations of symbiotic miR172c and local miR172c elicited by fixed nitrogen and development in leaves activate florigen-encoding *FLOWERING LOCUS T* (*FT*) homologs (*GmFT2a/5a*) by repressing *TARGET OF EAT1-like 4a* (*GmTOE4a*). Thus, FTs trigger reproductive development, which allows legumes to survive and reproduce under low-nitrogen conditions.

## Teaser

Symbiotic mobile miR172 and nitrogen systemically and synergistically promote flowering by activating FT-like genes.

## INTRODUCTION

Since ancient times, the importance of symbiotic nitrogen (N) fixation (SNF) by legumes has been noted. Two thousand years ago, it was recorded by a Roman scholar that planting legumes makes the soil more fertile for subsequent crop growth. In the same time period, a Chinese agronomist described the importance of nutrient-producing root organs (now defined as root nodules) for legume yield and warned that injury to root nodules can cause yield reduction in the earliest agronomic book of China, *The book of Fan Shengzhi*. It was not until the late 19th century that it was found that legumes exerted these miraculous roles through symbiotic association with nitrogen-fixing bacteria (rhizobia) of root nodules that can fix atmospheric nitrogen (*1*, *2*). Since then, research has mainly focused on the mechanisms underlying nitrogen-fixing nodule symbiosis. This led to many notable discoveries in a deep understanding of the fundamental mechanisms of legume-rhizobia symbiosis, how it forms and how it fixes nitrogen (*3*–*5*).

Under low-N (LN) conditions, legumes sense the levels of soil N and initiate the legume-rhizobium association by secreting flavonoid compounds (rhizobia) (*6*). Rhizobia recognize plant signals to synthesize and release lipochitooligosaccharides called Nod factor (NF) (*7*). NFs are perceived by the LysM-type NF receptors (NFRs) of legumes to trigger rhizobial infection and nodule formation (*8*–*10*). In soybean (*Glycine max*), GmNFR1α and GmNFR5α are indispensable for the recognition of rhizobial NFs and the initiation of rhizobial infection and nodule formation (*11*, *12*). Loss-of-function GmNFR1α mutants (e.g., *nod49*) fail to form symbiosis with *B. japonicum* (*11*). The symbiotic signals are then transmitted through the NF signaling pathway within root epidermal cells and between root cells/tissues. This pathway consists of calcium channels, nuclear calcium-calmodulin kinases, transcription factors, and other nodulation genes that work in concert to regulate two simultaneously occurring processes: infection and nodule organogenesis (*3*, *13*, *14*). After rhizobia are released into nodule cells, they become symbiosomes with plant membranes that differentiate into N-fixing bacteroids. Within root nodules, these nitrogen-fixing bacteria can convert atmospheric N_2_ to ammonia that the host plant can use in exchange for carbon and other nutrients from the plant (*15*, *16*).

In annual legumes such as soybean, rhizobia invade the plant root through root hairs. Root nodule initiation takes place in the outer cortex within the taproot, requiring nodule primordia and further growth to break through the epidermis before they emerge into the soil. A well-nodulated soybean plant usually has five to seven nodules on the taproot 2 weeks after emergence, and root nodules begin to fix nitrogen until plants with two to three fully expanded trifoliates. The number of nodules formed and the amount of nitrogen fixed in a nodulated soybean plant continue to increase during vegetative growth and reach a peak at the flowering stage before seed development begins (*17*). Thus, SNF in nodules is able to supply a majority of N to meet the nutrient requirement at the reproductive stage. Theoretically, SNF (e.g., nodulation and nitrogen supplies) and developmental timing (e.g., flowering time) interwine to affect plant reproductive growth, which determines the yield and quality of soybean. Whether and how symbiotic nodulation and nitrogen fixation are coordinated with developmental timing remain to be uncovered.

Flowering at the appropriate time is essential for plants to maximize their reproductive success and seed production (*18*–*20*). Flowering time is coordinately controlled by five main pathways, including endogenous/age, vernalization, photoperiod, gibberellins, and autonomous pathways, in Arabidopsis (*21*, *22*). The age pathway relies on the expression of two small noncoding RNAs and their target genes. microRNA156 (miR156) gradually decreases with age, while its target SQUAMOSA PROMOTER BINDING-LIKE (SPL) transcription factors increase. SPLs then activate the expression of the flowering promoter miR172, which promotes flowering by suppressing its flowering repressor target, APETALA2 (AP2) transcription factors (*23*, *24*). Flowering time is tightly linked to internal (e.g., genetic cues and nutrition) and external (e.g., light and temperature) information, and these signals converge on the transcriptional regulation of florigen FLOWERING LOCUS T (FT) (*21*, *22*), which then act as mobile flowering signals from the leaves to the shoot apical meristem to trigger the floral transition (*25*, *26*). For example, plants show a U-shaped flowering curve in response to different levels of nitrate. The optimal flowering time only occurs once the plants have acquired sufficient nitrate, while delayed flowering occurs under nitrate starved/limiting or superior conditions (*27*). In line with this, miR156 was up-regulated under nitrate-limiting conditions, while miR172 was down-regulated (*27*). In addition, the expression of the downstream gene *FT* was also repressed by high-N and LN conditions (*27*). Thus, miR156/miR172 have been proposed as a critical node that mediates cross-talk of the N signaling system with the age pathway to control the timing of flowering.

Current evidence has revealed that legumes share conserved flowering pathways (*28*–*30*). In soybean, miR156 and miR172 are crucial flowering regulators in the age flowering pathway. These two miRNAs showed a dynamic expression pattern with similarity to Arabidopsis miR156/miR172, and overexpression of miR156b and soybean *TARGET OF EAT1-like 4a* (*GmTOE4a*) genes, a target of miR172, delayed soybean flowering (*31*, *32*). Although the upstream regulators in the photoperiodic flowering pathway are quite different, the photoperiodic signal eventually converges to *FT* homologs (e.g., *GmFT2a* and *GmFT5a* in soybean) that control the timing of flowering (*33*–*38*). However, the number of these key flowering regulators is substantially increased in soybean, and their functions are markedly different (*28*, *33*, *34*, *39*). Our previous data showed that there are 12 members of miR172 in soybean and, among them, miR172c is a key nodulation promoter. miR172c has been shown to be important for rhizobia infection and nodule formation, as it is highly induced by rhizobia, and its expression progressively increases during nodulation, which can promote rhizobia infection and nodule formation (*40*, *41*). miR172c is also associated with flowering time, as overexpression of miR172 can accelerate flowering in Arabidopsis (*42*, *43*). Given that miR172c promotes both symbiotic nodulation and flowering, the role of miR172c in the integration of SNF and flowering needs to be uncovered to understand the emerging links between legume-rhizobial symbiosis, fixed nitrogen, and the onset of flowering.

In this study, we investigated the impact of symbiotic nodulation on the flowering time of legumes and the role of miR172c in inoculated soybean plants. Our findings demonstrate that SNF accelerates flowering in legumes. We found that symbiotically produced miR172c acts as a long-distance mobile signal moving from nodules to leaves, while nitrogen fixed in nodules induces leaf miR172c. These miR172c changes raise the total miR172 level that is necessary and sufficient for plant flowering. This suppresses transcriptional repression of the flowering repressor GmTOE4a on *GmFT*s, leading to accelerated flowering. In our study, we found a symbiosis pathway that regulates flowering time, affecting the reproductive success and yield of legumes.

## RESULTS

### SNF accelerates flowering

**Fig. 1.**
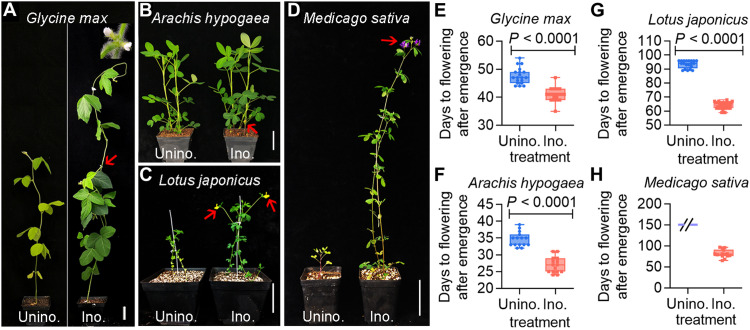
Symbiosis accelerates flowering of legumes under LN conditions . (**A** to **D**) Soybean (*G. max* cv. Jack) inoculated without or with *B. diazoefficiens* USDA110 (A), peanut (*A. hypogaea* cv. Yuanza 6) without or with *B. yuanmingense* CCBAU 45321 (B), birdsfoot trefoil (*L. japonicus* cv. MG-20) without or with *M. loti* MAFF303099 (C), and alfalfa (*M. sativa* cv. Gannong 9) without or with *S. meliloti* 2011 (D) under LN conditions when the first flower appeared. Scale bars, 5 cm. Red arrows indicate the first flowering buds. (**E** to **H**) Days from the emergence to flowering of the rhizobia uninoculated and inoculated with *G. max* (E), *A. hypogaea* (F), *L. japonicus* (G), and *M. sativa* (H). Data are the means ± SDs (*n* ≥ 15), and statistical significance was determined using Student’s *t* tests.

To investigate whether and how SNF influences flowering, we conducted experiments with four distantly related legume species: soybean (*G. max* cv. Jack), peanut (*Arachis hypogaea* cv. Yuanza 6), birdsfoot trefoil (*Lotus japonicus* cv. MG-20), and alfalfa (*Medicago sativa* cv. Gannong 9). We compared the flowering times of rhizobia-inoculated and uninoculated plants under long-day (16-hour light/8-hour dark) and LN conditions. Rhizobial inoculation substantially accelerated legume flowering in all species investigated. In soybean and peanut, rhizobial inoculation accelerated flowering by approximately 7 to 8 days (soybean, 6.7 ± 0.87; peanut, 7.7 ± 0.82; Fig. 1, A, B, E, and F, and fig. S1, A to H). In *L. japonicus*, the average flowering time was 29 ± 0.67 days earlier in inoculated plants than in uninoculated control plants (Fig. 1, C and G, and fig. S1, I to L), while in *M. sativa*, inoculated plants flowered at least 70 days earlier than the uninoculated control, which failed to flower because of nitrogen deficiency (Fig. 1, D and H, and fig. S1, M to P).

Pairwise inoculation comparisons confirmed that rhizobial symbiosis substantially accelerated flowering time for all tested legumes under LN conditions, but the degree to which this occurred differed across the species (Fig. 1, E to H). To investigate this further within a species, we assessed different cultivars of soybean [Williams 82 (W82) and Dongnong 50 (DN50)] and peanut [Zhonghua 24 (ZH24) and Huayu 36 (HY36)]. Each of these cultivars displayed significantly advanced flowering (by approximately 6 to 8 days) after being inoculated with rhizobia (W82, 7.9 ± 0.80; DN50, 7.5 ± 0.52; ZH24, 6.1 ± 0.86; HY36, 6.2 ± 1.2; figs. S2 and S3), further demonstrating that rhizobial symbiosis accelerates flowering similarly within the species.

### Nitrogen- and rhizobia-induced signals regulate flowering time

**Fig. 2.**
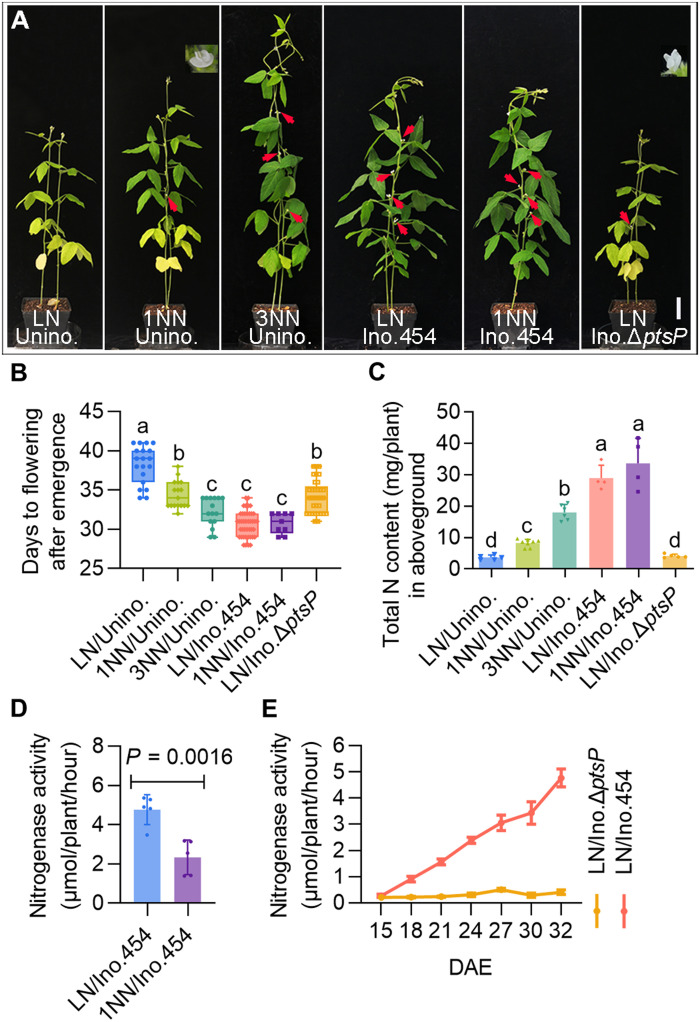
Nitrogen and symbiotic signals collaboratively promote flowering. (**A**) Phenotypes of the DN50 plants under different treatments at 32 days after emergence (DAE). Red arrows indicate flowering buds. Scale bar, 5 cm. These treatments include no rhizobia inoculation under LN, 1NN, and 3NN conditions (LN/Unino., 1NN/Unino., and 3NN/Unino.), rhizobial inoculation with *S. fredii* CCBAU 45436 under LN and 1NN conditions (LN/Ino.454 and 1NN/Ino.454), or with the mutant strain Δ*ptsP* under LN conditions (LN/Ino.Δ*ptsP*). (**B**) Days from the emergence to flowering of DN50 plants under the above treatments. (**C**) Total nitrogen content (in milligrams per plant) in the aboveground parts of the above different DN50 treatments at 32 DAE. Data are the means ± SDs (*n* ≥ 4), and one-way analysis of variance (ANOVA) with Tukey’s test was used for the statistical analysis (*P* ≤ 0.05). (**D**) Quantification of nitrogenase activity in the nodules of DN50 plants inoculated with CCBAU 45436 under LN and 1NN conditions. Data are the means ± SDs (*n* = 5), and statistical significance was determined using Student’s *t* tests. (**E**) Quantification of nitrogenase activity in the nodules of DN50 plants inoculated with CCBAU 45436 or Δ*ptsP* at various times. Data are means ± SDs (*n* ≥ 3).

LN and moderate nitrogen concentrations can postpone and promote flowering in plants, respectively (*27*). To test whether the accelerated flowering time of inoculated soybean plants is due to nitrogen concentration, we analyzed the flowering time of uninoculated soybean DN50 plants supplemented with LN thrice or LN twice with normal nitrogen once (1NN) or 3NN. The soybean plants flowered late upon nitrogen starvation, and plants receiving 1NN or 3NN flowered earlier than those grown under LN conditions (Fig. 2, A to C, and fig. S4). Similar trends were observed in the soil plant analysis development (SPAD) values and aboveground nitrogen content (Fig. 2C and fig. S4D). These data indicate that a nitrogen-controlled flowering pathway also exists in soybean.

To investigate the role of nitrogen application on the flowering time of inoculated plants, we investigated soybean plants inoculated with wild-type *Sinorhizobium fredii* CCBAU 45436 under LN and 1NN conditions. All the inoculated plants flowered almost at the same time, and the aboveground parts of these plants also had similar SPAD values and nitrogen contents (Fig. 2, A to C, and fig. S4). We then quantified the level changes of nitrogenase activity of the nodules from the plants grown under different nitrogen conditions. The nitrogenase activity of nodules from the plants grown under moderate nitrogen was much lower than that from inoculated plants under LN conditions (Fig. 2D), which is consistent with the well-known nitrogen inhibition of nitrogenase activity (*44*). Moreover, the flowering time of these inoculated plants with different levels of nitrogen fertilizer was comparable to that of the uninoculated plants with 3NN, although their aboveground nitrogen levels were higher (Fig. 2, A to C). Therefore, these results suggest that inoculated soybean develops a mechanism that integrates SNF and nitrogen signals to control the floral transition process.

To dissect the contributions of fixed nitrogen and symbiotic signals to flowering in inoculated plants, we inoculated DN50 under LN with wild-type *S. fredii* CCBAU 45436 or a Δ*ptsP* mutant of this strain, which does not have the functional nitrogen phosphotransferase system component EI^Ntr^ and produces ineffective nodules on soybeans (*45*). Plants inoculated with Δ*ptsP* formed ineffective nodules that had very low nitrogenase activity and did not fix nitrogen (Fig. 2, A, C, and E, and fig. S4). These Δ*ptsP*-inoculated plants with ineffective nodules flowered 3.5 ± 0.4 days later than wild-type rhizobia-inoculated plants but still flowered 4.0 ± 0.5 days earlier than the uninoculated control plants (Fig. 2B). These results strongly suggest that there is a symbiosis-specific regulatory pathway of flowering time in addition to the nitrogen flowering pathway of nodulated plants.

### Combined systemic and locally expressed miR172c can regulate flowering time

**Fig. 3.**
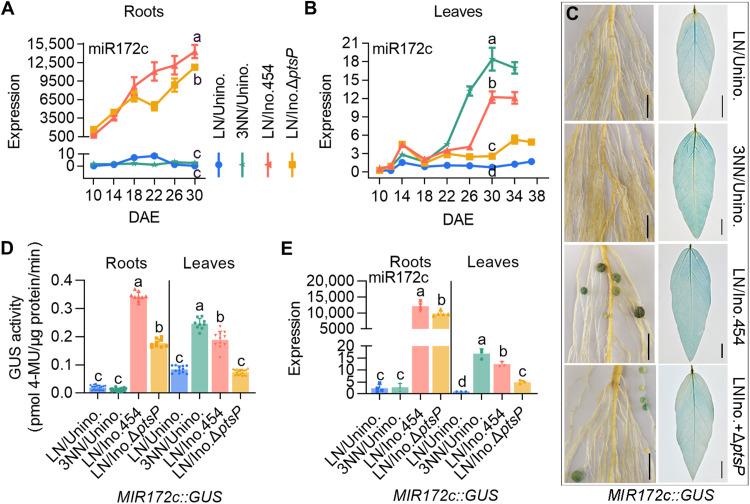
miR172c expression patterns in DN50 under different nitrogen and inoculation treatments. (**A** and **B**) miR172c expression patterns in DN50 roots (A) and leaves (B) of different treatments (LN and 3NN without rhizobial inoculation and LN with *S. fredii* CCBAU 45436 or the mutant strain Δ*ptsP*) at the specified time points. Data are means ± SDs (*n* = 3). (**C** and **D**) Representative images (C) and quantification (D) of *MIR172c*::GUS activity in the roots and leaves of *MIR172c::GUS* transgenic DN50 plants under the above different treatments at 32 DAE. Scale bars, 1 cm. Data are means ± SDs (*n* ≥ 9). (**E**) miR172c abundance in the roots and leaves of the *MIR172c::GUS* transgenic plants at 32 DAE. Data are means ± SDs (*n* ≥ 3). One-way ANOVA with Tukey’s test was used for the statistical analysis (*P* ≤ 0.05).

In legumes, a large number of symbiosis-specific miR172s are produced to promote nodule formation and rhizobia-legume symbiosis (*40*, *46*–*49*). Given that miR172 serves as a central regulator of flowering time in plants (*21*, *27*, *42*, *43*), we hypothesized that symbiotic miR172 is a key mediator of symbiosis-accelerated flowering. To test this hypothesis, we first quantified the spatial and temporal expression changes of miR172c, which is specifically expressed in soybean nodulation and increases progressively throughout nodule development until maturity (*40*), in both roots and shoots of uninoculated and inoculated DN50 plants. In roots of uninoculated plants under LN conditions, miR172c expression remained relatively stable at very low levels, while in roots of inoculated plants, miR172c levels progressively increased by more than a thousand-fold at 30 days after emergence (DAE) as flowering occurred (Fig. 3A). Similarly, miR172c transcript abundance remained at stable and low levels in leaves of uninoculated plants under LN conditions, whereas it exhibited an over 12-fold increase in leaves of inoculated plants at 30 DAE, peaking at the same time flowering occurred (Fig. 3B). These results show that the expression pattern of symbiotic-specific miR172c in roots correlates well with leaf miR172c abundance and flowering time.

To discriminate whether up-regulation of miR172c transcripts in inoculated roots and leaves is caused by fixed nitrogen and/or symbiotic signals, we measured the miR172c abundance in a time-course manner using DN50 plants inoculated with wild-type or mutant Δ*ptsP* strains or provided with increasing concentrations of nitrogen. The transcript levels of miR172c markedly increased in the roots of Δ*ptsP*-inoculated plants but were lower than those detected in wild-type strain–inoculated plants (Fig. 3A). The levels of miR172c abundance in leaves of Δ*ptsP*-inoculated plants were much lower than those in leaves of plants inoculated with the wild-type strain, and it was significantly decreased (approximately fourfold) in leaves of Δ*ptsP*-inoculated plants at 30 DAE (Fig. 3B). Failure to fix nitrogen by the Δ*ptsP* mutant strain may lead to the difference in miR172c abundance in leaves. In line with this, increased nitrogen (3NN) was sufficient to elevate the levels of miR172c abundance in leaves of uninoculated plants, and its abundance increased by more than 18-fold in leaves of plants with increased nitrogen at 30 DAE, although it did not induce the expression of miR172c in the roots (Fig. 3, A and B). However, the miR172c abundance in leaves of Δ*ptsP*-inoculated plants was still significantly higher (approximately 2.5-fold) than that in leaves of uninoculated plants at 30 DAE, suggesting that root systemic symbiotic miR172c may contribute to this. Together, our results suggest that both systemic symbiotic miR172c (roots) and nitrogen-induced miR172c (leaves) lead to increased miR172c abundance in leaves and early flowering.

To validate this, we generated DN50 transgenic plants expressing *MIR172c*:β-glucuronidase (*GUS*) and then monitored GUS activity and miR172c expression in the roots and leaves of plants under different nitrogen conditions or inoculated them with wild-type or Δ*ptsP* mutant strains. Almost no *MIR172c*::GUS activity was observed in uninoculated roots grown under either LN or 3NN conditions, but increased *MIR172c*::GUS activity was detected in leaves grown under 3NN conditions (Fig. 3, C and D), corresponding to the abundance of miR172c (Fig. 3E). This result suggests that nitrogen fertilization specifically induces miR172c expression in leaves. We further observed distinct profiles of *MIR172c*::GUS activity in inoculated plants. In roots, *MIR172c*::GUS activity was specifically present in both wild type– and Δ*ptsP*-inoculated nodules but not in the uninoculated control, although the latter had low basal GUS activity (Fig. 3, C and D), consistent with the level of miR172c (Fig. 3E), indicating that miR172c is mainly associated with nodules but not nitrogen. However, leaves of Δ*ptsP*-inoculated plants had *MIR172c*::GUS activity lower than that of wild-type strain–inoculated plants but similar to that of the uninoculated control under LN conditions (Fig. 3, C and D), although the abundance of miR172c in leaves of Δ*ptsP*-inoculated plants was higher than that of the uninoculated control under LN conditions (Fig. 3E). Thus, these data suggest that nodule-generated transmissible miR172c and leaf-expressed miR172c by nitrogen synergistically accelerate flowering.

### Nodulation-induced miR172c is a long-distance mobile signal

**Fig. 4.**
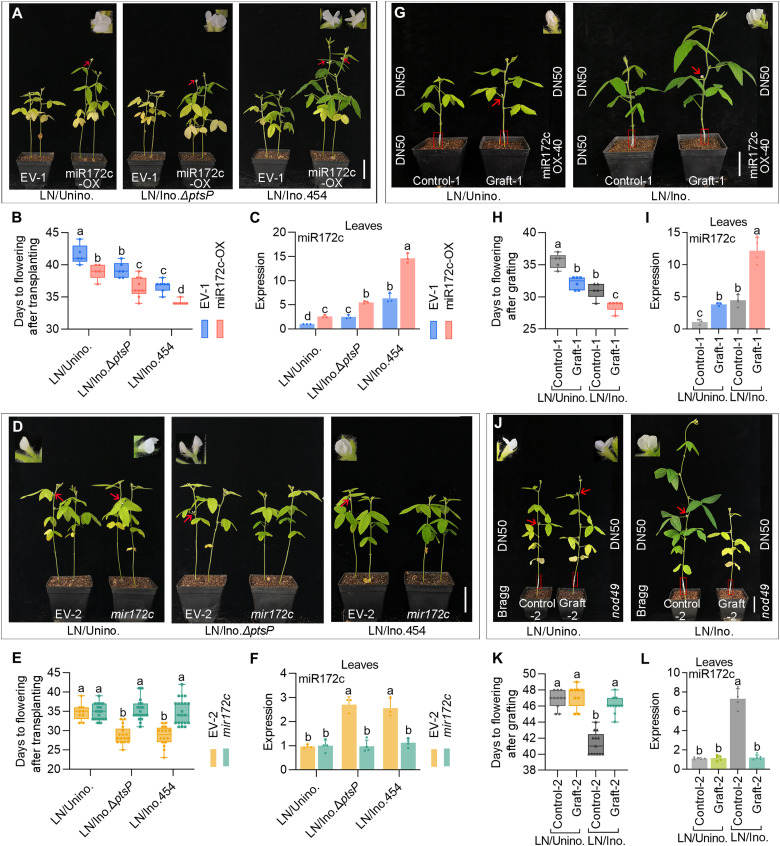
Long-distance transmission of symbiotic miR172c accelerates flowering. (**A**) Phenotypes of composite plants expressing EV-1 and 35S::miR172c (miR172c-OX) inoculated without or with *S. fredii* CCBAU 45436 or the mutant strain Δ*ptsP* under LN conditions. (**B**) Days from transplanting to flowering of EV-1 and miR172c-OX under the above treatments. Data are means ± SDs (*n* ≥ 5). (**C**) Quantitative reverse transcription polymerase chain reaction (qRT-PCR) analysis of miR172c expression in leaves of different EV-1 and miR172c-OX treatments. Data are means ± SDs (*n* = 3). (**D**) Phenotype of composite plants expressing EV-2 or CRISPR-Cas9 knockout *mir172c* roots inoculated without or with *S. fredii* CCBAU 45436 or mutant strain ΔptsP under LN conditions. (**E**) Days from transplanting to flowering of composite plants described in (D). Data are means ± SDs (*n* ≥ 10). (**F**) qRT-PCR analysis of miR172c abundance in leaves of the EV-2 and mir172c composite plants in (D). Data are means ± SDs (*n* ≥ 3). (**G** and **H**) Phenotypes and flowering time of self-grafted wild-type DN50 (left) and intraspecific grafts between a wild-type scion and stale miR172cOX-40 rootstock (right) inoculated without and with *B. diazoefficiens* USDA110 under LN conditions. Data are means ± SDs (*n* ≥ 5). (**I**) miR172c abundance in leaves of grafted plants in (G). Data are means ± SDs (*n* ≥ 3). (**J** and **K**) Phenotypes and flowering time of grafted plants with non-nodulating mutant nod49 root stock inoculated without or with *B. diazoefficiens* USDA110 under LN conditions. Data are means ± SDs (*n* ≥ 10). (**L**) miR172c abundance in leaves of grafted plants shown in (J). Data are means ± SDs (*n* ≥ 4). Photos were taken at appearance of the first flowering. For each phenotype photo, red arrows indicate the first flowering buds, and red boxes indicate grafted sites. Scale bars, 5 cm. One-way ANOVA with Tukey’s test was used for statistical analysis (*P* ≤ 0.05).

To determine whether miR172c acts as a long-distance signal, we evaluated the flowering time of DN50 plants consisting of wild-type shoots and transgenic hairy roots (composite plants) overexpressing miR172c (miR172c-OX) under LN conditions. Overexpression of miR172c in roots markedly promoted flowering of uninoculated composite plants (Fig. 4, A to C, and fig. S5, A and B). We then analyzed the miR172c levels in phloem exudates collected from freshly detopped seedlings without hypocotyls and cotyledons (detopped roots) or roots of uninoculated miR172c-OX and empty vector (EV) (EV-1) control composite plants. The exudates from detopped uninoculated miR172c-OX roots contained significantly higher levels of miR172c than those from detopped uninoculated vector control roots (fig. S6, A to C). These data suggest that root-to-shoot long-distance phloem transport of miR172c promotes flowering.

Next, we evaluated the flowering time of miR172c-OX and EV control composite plants that were inoculated with wild-type *S. fredii* CCBAU 45436 or the Δ*ptsP* mutant strains. All the *S. fredii* CCBAU 45436– or Δ*ptsP*-inoculated vector control composite plants flowered earlier than the uninoculated control, and both *S. fredii* CCBAU 45436– or Δ*ptsP*-inoculated miR172c-OX composite plants displayed a more severe early-flowering phenotype than their vector control plants. However, Δ*ptsP*-inoculated miR172c-OX and vector control composite plants flowered later than miR172c-OX and vector control composite plants inoculated with *S. fredii* CCBAU 45436 (Fig. 4, A to C, and fig. S5, A and B). The levels of leaf miR172c abundance and flowering time in these plants were correlated well with root miR172c (Fig. 4, A to C, and fig. S5, A and B). The difference in flowering time between Δ*ptsP*-inoculated and vector control composite plants was mainly due to systemic symbiotic miR172c, while the difference in flowering time between *S. fredii* CCBAU 45436– and Δ*ptsP*-inoculated composite plants was attributed to fixed nitrogen-induced miR172c. The earliest flowering of wild type–inoculated miR172c-OX composite plants is likely triggered by a combined rise in systemic miR172c (root overexpressed and symbiotic miR172c) and local nitrogen-induced miR172c. Analysis results of miR172c abundance in the phloem exudates collected from the detopped roots and roots of these inoculated EV control and miR172c-OX composite plants (fig. S6, A to C) support the notion that nodulation-induced miR172c moves from roots to shoots, triggering flowering.

To further validate the role of the symbiotic mobile miR172c in flowering time, we used CRISPR-Cas9 technology to knock out root miR172c (*mir172c*) and evaluated the flowering time of *mir172c* composite plants consisting of wild-type shoots and *mir172c* hairy roots (Fig. 4D and fig. S5, C and D). The miR172c knockout composite plants flowered later than their EV controls but similarly to their uninoculated vector and miR172c knockout plants regardless of inoculation with *S. fredii* CCBAU 45436, and the Δ*ptsP* strain was accompanied by lower miR172c abundance in the leaves upon miR172c knockout (Fig. 4, E and F). We also analyzed the miR172c levels in the phloem exudates collected from the detopped roots and roots of uninoculated and inoculated EV and *mir172c* composite plants. Upon miR172c knockout, levels of miR172c failed to show any increase in exudates collected from both detopped roots and roots of *S. fredii* CCBAU 45436– or Δ*ptsP*-inoculated plants compared with wild-type control plants (fig. S6, A, D, and E), supporting nodulation-specific miR172c as a mobile signal in triggering flowering.

Next, we generated a stable transgenic line, miR172c-OX-40, that flowered earlier (fig. S7) and performed grafting experiments using the stable transgenic line miR172c-OX-40 and wild-type plants. Grafted plants consisting of wild-type scions and miR172c-OX-40 rootstocks flowered earlier than the control plants with wild-type scions and rootstocks regardless of inoculation status (Fig. 4, G and H). Accordingly, miR172c expression in the leaves of grafted plants with miR172c-OX-40 rootstocks significantly increased (Fig. 4I). These results suggest that miR172c is transmitted from roots to leaves to promote flowering.

Previously, we showed that miR172c induction depended on the NFR GmNFR1α (*40*). To confirm that nodulation-specific miR172c movement to leaves triggers precocious flowering in soybean, we grafted the scions of uninoculated DN50 plants onto inoculated roots of Bragg or its *nod49* mutant, which is a non-nodulating mutant lacking functional GmNFR1α (*11*). Following grafting (Fig. 4, J to L), all uninoculated plants flowered similarly regardless of their rootstock and exhibited similarly low levels of miR172c expression in both roots and leaves (Fig. 4, J to L, and fig. S8). In contrast, inoculated plants having wild-type Bragg rootstocks flowered earlier and exhibited an increase in miR172c abundance in both roots and leaves (Fig. 4, J to L, and fig. S8). Inoculated plants with non-nodulating *nod49* mutant roots did not display the early-flowering phenotype and did not exhibit any increases in miR172c abundance in their roots or leaves (Fig. 4, J to L, and fig. S8). The combined results confirm that long-distance transmission of nodulation-specific miR172c and nitrogen fixation lead to increased leaf miR172c abundance and early floral induction.

### miR172c promotes flowering mainly through its target *TOE4a*

**Fig. 5.**
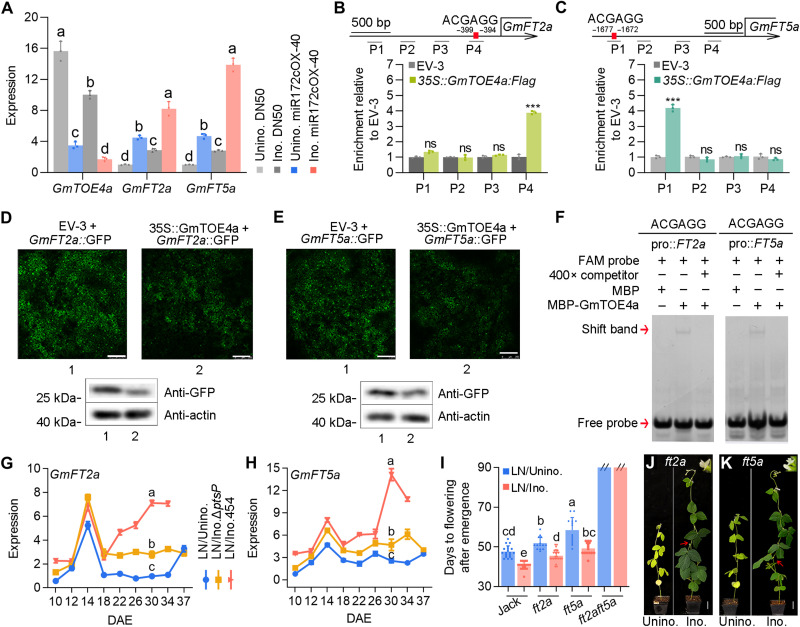
Symbiotic miR172c activates *GmFT2a/GmFT5a* to promote flowering by repressing *GmTOE4a*. (**A**) Expression of *GmTOE4a*, *GmFT2a*, and *GmFT5a* in the leaves of uninoculated and inoculated rhizobial DN50 and stable miR172c-OX-40 plants. (**B** and **C**) Chromatin immunoprecipitation (ChIP0) assay showing that GmTOE4a binds to the promoters of *GmFT2a* (B) and *GmFT5a* (C). P1 to P4 indicate four DNA fragments in the promoters. Data are means ± SDs (*n* = 3). Asterisks indicate the significance level at *P* ≤ 0.05 using Student’s *t* tests. ns, no significance. (**D** and **E**) GmTOE4a represses the promoter activity of *GmFT2a/GmFT5a*. Scale bars, 250 μm. (**F**) Electrophoretic mobility shift assay (EMSA) for detecting the GmTOE4a complex with the *GmFT2a* and *GmFT5a* probes. (**G** and **H**) The expression patterns of *GmFT2a* (G) and *GmFT5a* (H) in DN50 leaves under different treatments (LN without rhizobial inoculation and with *S. fredii* CCBAU 45436 or the mutant strain Δ*ptsP*). Data are means ± SDs (*n* = 3). (**I**) Flowering time of *ft2a*, *ft5a*, and *ft2aft5a* mutants without rhizobial inoculation and with inoculation of *B. diazoefficiens* USDA110. Data are means ± SDs (*n* ≥ 15). (**J** and **K**) Phenotypes of the *B. diazoefficiens* USDA110 uninoculated and inoculated *ft2a* (J) and *ft5a* (K) mutants. Red arrows indicate the first flowering buds. Scale bars, 5 cm. One-way ANOVA with Tukey’s test was used for the statistical analysis (*P* ≤ 0.05).

Multiple members of the miR172 gene family promote flowering by targeting AP2-like repressors, such as Target of EAT1 (TOE1) (*42*, *43*). To investigate whether miR172c exerts its function by targeting *TOE1* ortholog genes (*32*, *40*), we analyzed the expression of these genes in uninoculated and inoculated DN50 and miR172c-OX-40 plants at flower initiation (fig. S9). *GmTOE4a/4b* expression decreased notably in inoculated wild-type plants, showing the opposite expression pattern as miR172c (fig. S9), and *GmTOE4a* expression was down-regulated in the miR172c-OX-40 plants and was much lower after inoculation (Fig. 5A and fig. S9). Because *GmTOE4a* is a key repressor of flowering under long-day conditions (*32*), we hypothesized that *GmTOE4a* may be the target gene for miR172c-mediated nodulation-activated flowering. GmTOE4a is a typical AP2 family protein and is localized in the nucleus (fig. S10). To validate the relationship between miR172c and *GmTOE4a* (fig. S11A), we expressed *35S::GmTOE4a:GFP* (fig. S11B) alone or coexpressed it with 35S::miR172c in leaves of *Nicotiana benthamiana*. The transcript levels of GmTOE4a were markedly reduced by miR172c (fig. S11, B and C). Consistent with earlier RACE (Rapid amplification of cDNA ends) results (*32*), these findings suggest that *GmTOE4a* might be a main target of miR172c in nodulation-accelerated flowering.

### *GmTOE4a* controls the flowering of inoculated plants via *GmFT2a* and *GmFT5a*

In Arabidopsis, TOEs prevent premature flowering by directly repressing *FT*, which is a flowering activator (*50*, *51*). In soybean, *GmTOE4a* controls flowering time by down-regulating both *GmFT2a* and *GmFT5a* (*32*). To test whether the levels of *GmFT2a* and *GmFT5a* expression are directly regulated by GmTOE4a, we analyzed the promoters of the two genes. AP2 binding sites were identified in the promoters of both *GmFT2a* and *GmFT5a* (Fig. 5, B and C). We performed expression assays by coexpressing *35S::GmTOE4a* with either *GmFT2a::GFP* or *GmFT5a::GFP* in *N. benthamiana*. Findings from these studies revealed that GmTOE4a effectively reduced the transcription of both *GmFT2a* and *GmFT5a* (Fig. 5, D and E). Transient chromatin immunoprecipitation (ChIP) assays were subsequently performed in *N. benthamiana*. The *GmFT2a* and *GmFT5a* promoters were coimmunoprecipitated with GmTOE4a (Fig. 5, B and C), suggesting an interaction between the GmTOE4a and *GmFT2a/5a* promoters. An electrophoretic mobility shift assay (EMSA) confirmed the direct binding of GmTOE4a to the *GmFT2a* and *GmFT5a* promoter regions in vitro (Fig. 5F). These data demonstrate that GmTOE4a is a repressor of both *GmFT2a* and *GmFT5a*.

On the basis of the aforementioned findings, we hypothesized that nodulation- and nitrogen-induced miR172c in leaves may relieve the repression of *GmFT2a* and *GmFT5a* transcription by GmTOE4a. To test this hypothesis, we analyzed the expression of *GmFT2a* and *GmFT5a* in leaves of DN50 inoculated without and with wild-type *S. fredii* CCBAU 45436 or the Δ*ptsP* mutant strains. *GmFT2a* and *GmFT5a* expression similarly peaked at 14 DAE in both uninoculated and wild type– or Δ*ptsP*-inoculated plants, but their expression showed a distinct pattern thereafter (Fig. 5, G and H). The expression of both *GmFT2a* and *GmFT5a* genes in wild-type *S. fredii* CCBAU 45436–inoculated plants was then up-regulated and reached the highest levels at the earliest at 30 DAE when flowered; *GmFT2a* and *GmFT5a* in uninoculated plants had the lowest expression levels, while the expression levels of both genes in the Δ*ptsP*-inoculated plants were in between at the time (Fig. 5, G and H). The *GmFT2a* and *GmFT5a* expression levels correlated well with the timing of flowering of inoculated plants. Thus, both nodulation and nitrogen contributed to elevated *GmFT2a* and *GmFT5a* expression levels and shifted the peak of *GmFT2a* and *GmFT5a* expression at the second stage. miR172c overexpression enhanced the expression patterns of *GmFT2a* and *GmFT5a* (Fig. 5A and fig. S12, A and B), while the specific *GmFT2a* and *GmFT5a* expression patterns were disturbed in grafted *nod49* and *mir172c* mutant plants (fig. S12, C to F). All these results suggest that *GmFT2a* and *GmFT5a* are required for mediating nodulation-accelerated early flowering.

To demonstrate the redundant roles of *GmFT2a* and *GmFT5a* in nodulation-induced early flowering, we analyzed the flowering times of *ft2a* and *ft5a* single mutants and the *ft2aft5a* double mutant with and without rhizobial inoculation. Uninoculated *ft2a* and *ft5a* mutants both showed delayed flowering compared with the uninoculated wild-type plants, with the *ft5a* mutant displaying a more severe phenotype than *ft2a* (Fig. 5, I to K). Upon inoculation, *ft2a* and *ft5a* plants both flowered earlier than the uninoculated control plants but exhibited later flowering than the inoculated wild-type plants (Fig. 5I). The *ft2aft5a* double mutant did not flower after 90 DAE without inoculation, which is consistent with previous results (*35*). The double mutant completely lost its flowering response to rhizobial inoculation over a time period of 90 days (Fig. 5I). These results demonstrate that *GmFT2a* and *GmFT5a* are partially redundant genes required for nodulation-induced early flowering. Together, these results support the idea that symbiotic nodulation-mediated early flowering induction in soybean depends on *GmFT2a* and *GmFT5a*.

## DISCUSSION

**Fig. 6.**
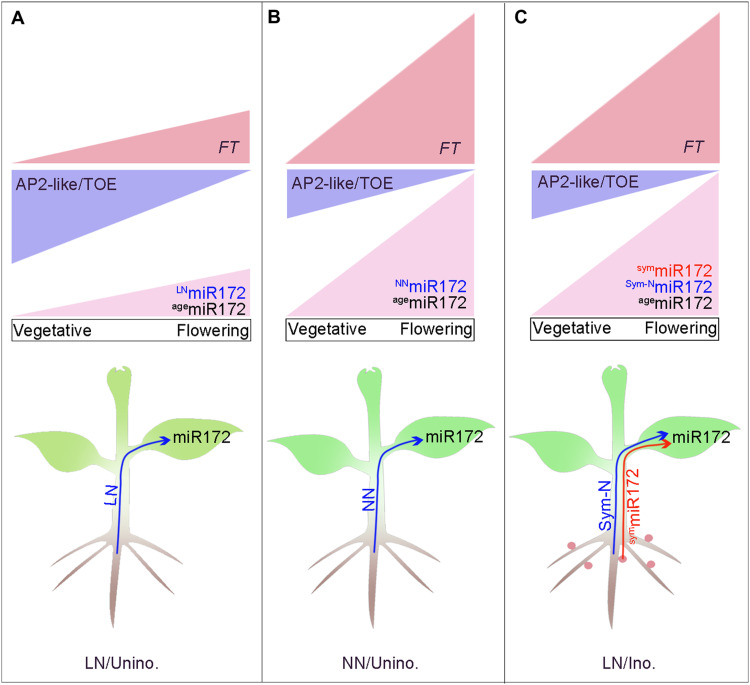
Legume-rhizobia symbioses accelerate flowering time via a mobile symbiotic miRNA and nitrogen. (**A**) In the absence of rhizobia under LN conditions, miR172s (^age^miR172 and ^LN^miR172) gradually increase during growth and promote flowering by activating the flowering-promoting homologs of *FT*s through reducing transcriptional repression of TOE when miR172s reach a peak under LN conditions. (**B**) In the absence of rhizobia under normal nitrogen conditions (NN), miR172s (^NN^miR172 and ^age^miR172), which are induced in leaves by nitrogen (^NN^miR172), and age-related miR172 (^age^miR172) cause an early peak in leaf miR172 abundance. miR172s reduce the abundance of the floral repressors AP2-like/TOEs and activate the flowering integrator *FT*s, thereby triggering early flowering. (**C**) Upon rhizobial inoculation, miR172s (^age^miR172, ^Sym-N^miR172, and ^sym^miR172), which are induced in leaves by fixed nitrogen (^Sym-N^miR172) and in roots induced and transmitted from roots to leaves (^sym^miR172), and age-related miR172 (^age^miR172) in leaves caused an early peak in leaf miR172 abundance. miR172s reduce the abundance of the floral repressors AP2-like/TOEs and activate the flowering integrator *FT*s, thereby triggering early flowering. Blue lines represent nitrogen transport to leaves, and red lines indicate miR172 moving from roots to leaves.

SNF and flowering time are two major determinants of yield in soybean (*18*–*20*, *52*), and the genetic relationship between two agriculturally important traits and the underlying molecular mechanism remain elusive. In this study, we demonstrated that nodulation and available nitrogen can accelerate the flowering of legumes and that an integrated flowering pathway exists to systemically control flowering in legumes. Our findings show that miR172c levels are systemically up-regulated in leaves by rhizobial symbiosis and N inputs in roots. The elevated levels of miR172c in the leaf target transcripts of *GmTOE4a* for degradation. GmTOE4a is a repressor of both *GmFT2a* and *GmFT5a*, and hence, its degradation leads to an up-regulation in GmFT2a and GmFT5a levels, which subsequently results in floral initiation. Thus, both rhizobial symbiosis and nitrogen availability pathways are integrated and coordinated in the leaf via miR172c to induce floral initiation, and this occurs by repressing GmTOE4a, which activates *GmFT2a* and *GmFT5a* to trigger florigen production and accelerate flowering (Fig. 6 and fig. S13). Overall, we proposed that miR172c may be a morphogen that specifies cell fates and floral initiation in a concentration-dependent manner by inversely regulating its target gene expression. Soybeans have multiple miR172 members that have distinct expression patterns (*40*). Although we have previously shown that miR172c regulates nodulation, nodule number, and salt tolerance by targeting nodule number control 1 (NNC1) (*40*, *53*), how these miR172s integrate genetic and environmental cues into developmental programs through different target genes to confer developmental plasticity is an interesting question for future research.

In *L. japonicus*, *Medicago truncatula*, and common bean, miR172a and miR172c, orthologs of soybean miR172c (fig. S14A), mediate nodulation (*47*–*49*). We analyzed the miR172 expression patterns and found that symbiotic-induced miR172s were also correlated with the miR172 peaks in leaves and the timing of flowering of inoculated *L. japonicus* and *M. sativa* plants (fig. S14, B to E). We propose that the miR172-centered nodulation and nitrogen flowering pathway are conserved in legumes. Because the roles of miR172s in nodulation and miR172/TOE/FT-regulated flowering are highly conserved in legumes (*28*, *32*, *35*–*37*, *40*, *46*–*49*, *54*), it is likely that similar pathways exist among leguminous plant species that encourage flowering under nodulation or ample N availability. Thus, the miR172-TOE-FT module could be a key module for screening and breeding elite legume varieties with optimized flowering times and high seed yields for various growing conditions. These findings may also have important agricultural and ecological implications in reducing fertilizer overuse and its adverse effects on biodiversity and climate change.

## MATERIALS AND METHODS

### Legume cultivars and mutants

The soybean cultivars W82, DN50, Bragg, and Jack were used in this study. Bragg and *GmNFR1a* mutant *nod49* were generated by P. M. Gresshoff (The University of Queensland, Australia). Peanut cultivars Yuanza 6, ZH24, and HY36 were provided by B. Wang (Huazhong Agriculture University, China). Seeds of *L. japonicus* ecotype Miyakojima MG-20 and *M. sativa* cultivar Gannong 9 were provided by D. Duanmu (Huazhong Agriculture University, China) and R. Zhu (Institute of Grass Industry, Heilongjiang Academy of Agricultural Sciences, China), respectively.

The *ft2a*, *ft5a*, and *ft2aft5a* single and double mutants in the Jack background were obtained by *Agrobacterium*-mediated transformation using CRISPR-CAS9 technology (*35*). Transgenic plants miR172c-OX and expressing *MIR172c::GUS* were obtained by *Agrobacterium*-mediated transformation as described below in the plasmid construction and transformation section.

### Rhizobium strains

*Bradyrhizobium diazoefficiens* USDA110, *S. fredii* CCBAU 45436, and the Δ*ptsP* mutant in its background were used for inoculating soybean cultivars. *Bradyrhizobium yuanmingense* CCBAU 45321 inoculated with peanut was provided by X. Sui (China Agriculture University). *Mesorhizobium loti* MAFF303099 and *Sinorhizobium meliloti* 2011 used for inoculating *L. japonicus* cv. MG-20 and *M. sativa* cv. Gannong 9 were provided by D. Duanmu and Y. Cao (Huazhong Agriculture University, China), respectively.

### Plant growth and rhizobia inoculation

Various legume seeds were planted in autoclaved vermiculite in pots (7 cm by 7 cm and 10 cm by 10 cm). After that, the plants were irrigated alternately with water and Broughton and Dilworth LN (0.25 mM nitrate) nutrient solution [CaCl_2_ (0.128 g/liter), Ca(NO_3_)_2__·_4H_2_O (0.03 g/liter), KH_2_PO_4_ (0.068 g/liter), C_6_H_5_O_7_Fe (0.00245 g/liter), MgSO_4__·_7H_2_O (0.0616 g/liter), K_2_SO_4_ (0.0435 g/liter), MnSO_4__·_H_2_O (0.1690 mg/liter), H_2_BO_3_ (0.1237 mg/liter), ZnSO_4__·_7H_2_O (0.1438 mg/liter), CuSO_4__·_5H_2_O (0.0499 mg/liter), CoSO_4__·_7H_2_O (0.0281 mg/liter), and Na_2_MoO_4__·_2H_2_O (0.024 mg/liter)] or normal nitrogen nutrient solution (7.88 mM nitrate) [Ca(NO_3_)_2__·_4H_2_O (0.2357 g/liter), KH_2_PO_4_ (0.068 g/liter), C_6_H_5_O_7_Fe (0.00245 g/liter), MgSO_4__·_7H_2_O (0.0616 g/liter), K_2_SO_4_ (0.0435 g/liter), (NH_4_)_2_SO_4_ (0.3884 g/liter), MnSO_4__·_H_2_O (0.1690 mg/liter), H_2_BO_3_ (0.1237 mg/liter), ZnSO_4__·_7H_2_O (0.1438 mg/liter), CuSO_4__·_5H_2_O (0.0499 mg/liter), CoSO_4__·_7H_2_O (0.0281 mg/liter), and Na_2_MoO_4__·_2H_2_O (0.024 mg/liter)] (*55*). During the plant culture, a total of three nutrient solutions were irrigated, and each seedling was irrigated with 70 ml of nutrient solution each time. Under LN conditions, the plants were watered three times with LN nutrient solution during the test period; under 1NN conditions, the plants were first watered twice with LN nutrient solution, followed by the normal nitrogen nutrient solution once; Under 3NN conditions, the plants were irrigated three times with normal nitrogen nutrient solution during the test period. As described previously, the plants were grown in a growth house (16-hour light/8-hour dark, 25°C, 50% relative humidity). The rhizobia were cultured with Tryptone-Yeast (TY) medium in a 28°C shaker (*40*).

When sowing, the noninoculated plants were watered with distilled water, while the inoculated plant seedlings were inoculated with the corresponding rhizobium suspended in distilled water. Each soybean seedling was inoculated with 30 ml of *B. diazoefficiens* USDA110 [optical density at 600 nm (OD_600_) = 0.08], *S. fredii* CCBAU 45436 (OD_600_ = 0.08), or Δ*ptsP* mutant (OD600 = 0.08). Each peanut seedling was inoculated with 50 ml of *B. yuanmingense* CCBAU 45321 (OD_600_ = 0.08). Each *L. japonicus* MG-20 seedling was inoculated with 10 ml of *M. loti* MAFF303099 (OD_600_ = 0.05). Each *M. sativa* cultivar Gannong 9 seedling was inoculated with 10 ml of *S. meliloti* 2011 (OD_600_ = 0.05).

### Determination of plant dry weight and total nitrogen content

The aboveground parts (shoots) of DN50 plants were taken at specified time points. Their dry weight was estimated after oven drying at 65°C to constant weight. The dried samples were used for the determination of total nitrogen in plant materials using the Kjeldahl method (Wuhan Triploid Biotechnology Co. Ltd.).

### Measurements of chlorophyll concentration

The total chlorophyll concentration (via approximation by SPAD) was determined on the fully expanded youngest leaves of soybean cultivar DN50 plants with a portable chlorophyll meter (SPAD-502, Minolta Sensing). At least five DN50 plants per treatment were measured, and three SPAD values per leaf were averaged as the mean SPAD value of the leaf.

### Plasmid construction

For the miR172c-pEGAD construct used in the hairy root experiment, the miR172c precursor sequence [220 base pairs (bp)] was cloned and inserted into the plant expression vector pEGAD (EV-1) as we described previously (*40*). For 35S::miR172c stable plant transformation, the miR172c precursor (220 bp) was amplified and inserted into a plant binary vector PTF101 using Age I and Eco RI. The miR172c-PTF101 construct was transformed into *Agrobacterium tumefaciens* strain EHA101 and used for DN50 transformation using the *Agrobacterium*-mediated cotyledon-node transformation method (*56*).

For *MIR172c::GUS* stable transformation, the promoter region 2640-bp upstream of the miR172c (MI0010727) start codon was cloned into the PTF102 vector containing the *GUS* reporter gene using Bam HI. The PTF102 vector contains the *Bar* gene conferring glufosinate resistance in plants. The resulting plasmid harboring *MIR172c*::*GUS* was used to transform DN50 plants using the *Agrobacterium*-mediated cotyledon-node transformation method (*56*).

For *GmTOE4a* overexpression, the constructs of *GmTOE4a*-pEG100 and *GmTOE4a*-pMDC83 were made. The pEG100 vector (EV-3) contains 3× FLAG at the C terminus, and the pMDC83 vector contains the *GFP* gene. The coding DNA sequence of *GmTOE4a* was amplified from W82 and cloned into pDONR207 by a recombination reaction between an attB DNA sement and an attP donor vector (BP) (Gateway entey clone) for sequencing, in which positive plasmids (pDONR207 with the *GmTOE4a* coding DNA sequence) were used to generate the constructs of *GmTOE4a*-pEG100 and *GmTOE4a*-pMDC83 by the a reaction between the attL sites of the entry clone and the attR sites of destination vector (LR) (Gateway expression clone).

For the activity analysis of *GmFT2a* and *GmFT5a* promoters, putative promoter regions of 2161-bp upstream of the *GmFT2a* start codon and 2121-bp upstream of the *GmFT5a* start codon were amplified from W82 genomic DNA and cloned into pDORNOR207 by the BP reaction for sequencing, in which positive plasmids (pDORNOR207 with the *GmFT2a* and *GmFT5a* promoter sequence) were used to generate the constructs of *GmFT2apro-*pMDC107 and *GmFT5apro-*pMDC107 by the LR reactions. pMDC107 vector containing the *GFP* reporter gene.

To knock out miR172c in DN50 cells, CRISPR-Cas9 technology was used. First, the precursor of miR172c was analyzed using the online software CRISPR-P (http://crispr.hzau.edu.cn/CRISPR2/), and the two most reliable single-guide RNAs (sgRNAs), TCAAGATTCCCATAGCAAAAGGG and TCAAGATTCACA AGCTTTAGGGG, were selected. Then, two *AtU6 promrters-sgRNA-AtU6 terminator* cassettes were amplified using the vector *pCBC-DT1T2* as a template. Next, the two fragments were cloned by the Golden Gate reaction into two *Bas* I sites of the backbone vector pKSE401–green fluorescent protein (GFP; EV-2) and transformed into chemically competent *Escherichia coli* DH5α (*57*). The miR172c-CRISPR-Cas9 construct was validated by sequencing, and the plasmids were then transformed into *Agrobacterium rhizogenes* strain K599 for hairy root transformation. All of the primers used for plasmid construction are listed in table S1.

### Soybean hairy root transformation and *B. diazoefficiens* inoculation assay

*A. rhizogenes* strain K599 containing a specific vector was used for soybean hairy root transformation as described previously (*40*). The transgenic composite plants (composite plants) were transferred to small pots (10 cm by 10 cm) containing vermiculite and grown (16-hour light/8-hour dark, 25°C, 50% relative humidity). For the overexpression of miR172c (miR172c-OX) or an EV (EV-1)–transformed soybean hairy root composite plants, each plant was inoculated without or with 30 ml of *S. fredii* CCBAU45436 and Δ*ptsP* mutant (OD_600_0 = 0.08) suspended in distilled water at 10 days after transplanting.

For the transgenic hairy roots transformed with pKSE401-GFP EV (EV-2) and premiR172c-pKSE401-GFP (CRISPR-Cas9 technology to knock out miR172c), roots were screened using a portable fluorescence lamp (LUYOR-3415, LUYOR, USA) to visualize GFP green fluorescence at 7 days after transplanting. The untransformed roots (does not have green fluorescence) were cut and then transplanted again. Each plant was inoculated without or with 30 ml of *S. fredii* CCBAU45436 and Δ*ptsP* mutant (OD_600_ = 0.08) suspended in distilled water at 5 days after retransplantation. Water and Broughton and Dilworth LN nutrient solution were irrigated alternately during the growth of plants (*55*).

### RNA extraction and expression analysis

Samples were taken 4 hours after the start of the photoperiod. Total RNA and small RNAs were extracted from the samples of plant leaves and roots (without or with nodules) using TRIzol reagent (Aidlab Biotechnologies Co. Ltd., Beijing, China). Total RNA was treated with genomic DNA Wiper Mix (Yeasen Biotech, Shanghai) to remove genomic DNA. cDNA strands were synthesized from the RNAs using a Hifair II first Strand cDNA Synthesis SuperMix for quantitative polymerase chain reaction (qPCR) kit (Yeasen Biotech, Shanghai). qPCR was performed using a Hieff qPCR SYBR Green Master Mix kit (Yeasen Biotech) with gene-specific primers (table S1). *GmELF1b* was used as an internal control.

Stem loop–specific RT for miRNA in soybean, *L. japonicus* and *M. sativa* was performed as described previously (*40*). MiR1520d was used as an internal control of miRNA in soybean (*40*). U6s were used as internal controls of miRNA in *L. japonicus* and *M. sativa* (*47*). Quantitative reverse transcription PCR (qRT-PCR) was conducted using a Hieff qPCR SYBR Green Master Mix kit (Yeasen Biotech) with the gene-specific primers listed in table S1.

### Identification of transgenic soybean plants

The putative stable transgenic soybean plants miR172c-OX and expressing *MIR172c::GUS* were first screened by leaf painting with 0.8% glufosinate solution. The glufosinate-resistant plants were then confirmed by PCR. Genomic DNA was extracted from the leaves of glufosinate-resistant lines to detect the *Bar* gene using the primers listed in table S1. The plants that were glufosinate-resistant and contained the *Bar* gene were further used for the identification of stable transgenic plants. For the miR172c-OX lines, the expression levels of miR172c were analyzed. For the *MIR172c::GUS* lines, GUS histochemical staining was performed to identify the lines expressing *MIR172c::GUS*.

For identification of hairy root transformants, first, PCRs were performed to detect the *Bar* gene in the hairy roots transformed with *A. rhizogenes* strain K599 containing pEGAD EV (EV-1) and 35S::miR172c, and then the expression levels of miR172c were quantified by stem-loop RT-PCR as described above (*40*). Transgenic hairy roots were transformed with pKSE401-GFP EV (EV-2) or *premiR172c*-pKSE401-GFP, and the CRISPR-Cas9–mediated miR172c knockout vector was screened using a portable fluorescence lamp (LUYOR-3415, LUYOR, USA) to visualize GFP green fluorescence. The genomic sequence of premiR172c was amplified from individual transgenic hairy roots and validated with the specific primers miR172c-CrisperID (table S1). Types of gene editing were determined by sequencing. All of the primers used for the identification of transgenic soybean plants are listed in table S1.

### Plant grafting

The grafting method was modified according to a previous description (*58*). Briefly, soybean seedlings at 7 days after sowing were selected to cut the scion with a blade, which was trimmed into a wedge-shaped end. A bamboo toothpick was inserted into the young stem of the stock to make a hole approximately 1.5 to 2 cm in depth. The wedge-shaped scion was gently inserted into the hole of the treated root stock, and the root stock and scion were tightly stitched together to form a whole. The joint zone of the grafting plant was wrapped with parafilm (Beamis, Neenah, WI, USA) approximately 4 to 5 cm in length. The grafted seedlings were grown in a greenhouse (16-hour light/8-hour dark, 25°C, 50% relative humidity). For rhizobial inoculation, each plant was inoculated with 30 ml of *B. diazoefficiens* USDA110 (OD_600_ = 0.08) at 5 days after grafting.

### Collection of phloem exudates

Over six transgenic composite plants expressing 35S::miR172c and control EV-1 were watered at the night of 34 DAT (days after transfer), and *mir172c* and control EV-2 were watered at the night of 26 DAT. Phloem exudate collection was performed the next day.

For the collection of phloem exudate flowing from underground roots to the aboveground parts through the phloem, the stems (1 to 2 cm above the transition zone between the root and the aboveground part of the soybean) were chosen as the collection site. Specifically, the shoots of the soybean plants were cut off 1 to 2 cm above the transition zone between the roots and the shoots to obtain detopped roots. The cut surfaces were immediately cleaned three times with sterile filter paper and 20 mM K_2_-EDTA solution after cutting as described previously (*59*). Next, the exudates were immediately transferred to a reaction tube containing TRIzol using a pipettor.

For root (without or with root nodules) exudate collection, sterilized blades were used to cut off the roots. The roots were immediately placed in dishes containing 20 mM K_2_-EDTA. After three washes, the roots were immediately transferred into a reaction tube containing 20 mM K_2_-EDTA solutions and placed in the dark at 4°C overnight. The roots were removed the next day, and RNA was extracted from exudates using the TRIzol method (*60*).

### GUS staining and GUS activity assays

To examine the tissue-specific activity of the miR172c promoter, roots (without or with root nodules) and leaves of the stable transgenic *MIR172c*::GUS plants were collected for GUS staining. GUS staining was performed as described previously (*61*) with a few modifications. Briefly, samples were taken 4 hours after the start of the photoperiod at 32 DAE, and the leaves and roots were placed in GUS solution [50 mM phosphate buffer (pH 7.0), 0.25% Triton X-100, 5-bromo-4-chloro-3-indoxyl-β-d-glucuronide cyclohexylammonium salt (X-Gluc; 0.5 mg/ml; GoldBio, St. Louis, USA), and dimethyl sulfoxide (DMSO; 1 mg of X-Gluc in 5 μl of DMSO)], vacuumed for 10 min, then incubated in staining buffer for 5 to 6 hours for leaves at 37°C, 20 to 21 hours for roots at 37°C, and then washed three times with 75% ethanol.

For the fluorometric GUS assay, roots (without or with root nodules) and leaves of stable transgenic *MIR172c*::*GUS* plants were used to determine GUS enzyme activity, and GUS activity was quantified by measuring the rate of 4-methylumbelliferyl-β-d-glucuronide conversion to 4-MU (4-methylumbelliferone) according to a published procedure (*61*). β-Glucuronidase activity was determined using a SpectraMax i3x MultiMode Microplate Reader at emission at 455 nm as the excitation wavelength at 365 nm. Protein concentrations of the tested tissues were assessed by the Bradford method using a Quick Start Bradford 1× Dye Reagent Protein Assay (Bio-Rad, CA, USA). GUS enzyme activity was expressed as picomoles of 4-MU generated per minute per milligram of protein.

### Measurement of nitrogenase activity

The nitrogenase activity of root nodules was indirectly determined by the acetylene reduction assay (*62*). Because of the difficulty in isolating the small nodules, nitrogenase activity per plant was analyzed. Briefly, the roots with root nodules per plant were collected and put into a 50-ml vial with a flanged rubber septum. Air (2 ml) was withdrawn from the vial and replaced with an equal volume of acetylene. After incubation for 2 hours at 28°C, the amount of ethylene produced was measured by gas chromatography (GC-4100; EWAI, China). The nitrogenase activity of nodules was expressed as micromoles of ethylene generated per plant per hour.

### Subcellular localization of GmTOE4a and validation of posttranscriptional repression of GmTOE4a by miR172c

*A. tumefaciens* GV3101 containing the following binary vectors was cultured overnight. For transiently expressing one gene or coexpressing two genes, each culture alone or a mixed culture with an equal volume (1:1) of each culture was used for injection (the final OD_600_ was 0.3 for each).

To assess the subcellular localization of GmTOE4a, pMDC83-*TOE4a*-*GFP* was introduced into *A. tumefaciens* GV3101. Transient expression assays were performed in *N. benthamiana* leaves as described previously (*40*). At 2 days after cocultivation, the GFP and 4′,6-diamidino-2-phenylindole fluorescence of the infected tobacco leaves were observed with a Leica SP8 confocal microscope.

To validate the direct inhibition of *GmTOE4a* by miR172c, the constructs harboring 35S::miR172c and *35S::GmTOE4a:GFP* were transformed individually or cotransformed into *N. benthamiana* leaves, and the GFP signals of the tobacco leaves were observed with a Leica SP8 confocal microscope at 2 days after injection. The leaf materials were then collected for detection of GmTOE4a:GFP proteins using anti-GFP and anti-actin antibodies using immunoblot.

### Transcriptional activity analyses of GmTOE4a in *N. benthamiana* leaves

To assess the repression of *GmFT2a* and *GmFT5a* by GmTOE4a, the promoters of *GmFT2a* or *GmFT5a* were cloned into pMDC107 to generate the reporter constructs *GmFT2a::GFP* and *GmFT5a::GFP*. For the *35S::GmTOE4a* construct, the coding sequence of *TOE4a* was amplified and inserted into pEG100. The designated constructs were transformed alone or cotransformed into *N. benthamiana* leaves. Transient expression assays were performed in *N. benthamiana* leaves as described previously. At 2 days after injection, GFP fluorescence in the transformed *N. benthamiana* leaf cells was detected with a Leica SP8 confocal microscope, and the leaf materials were then collected for Western blotting to analyze the GFP proteins using anti-GFP and anti-actin antibodies.

### ChIP-qPCR assay

The designed constructs (pEG100/GmTOE4a-pEG100 + *GmFT2a/5apro*-pMDC162) were cotransformed into *N. benthamiana* leaves. Transient expression assays were performed in *N. benthamiana* leaves as described previously (*40*). One gram of *N. benthamiana* leaves containing pEG100 + *GmFT2a/5apro*-pMDC162 and GmTOE4a-pEG100 + *GmFT2a/5apro*-pMDC162 were used for the ChIP assay. The ChIP-qPCR assay was performed as described previously (*40*). The leaves were cross-linked with 1% formaldehyde for 30 min under vacuum; cross-linking was stopped with 0.125 M glycine. The leaves were ground in liquid nitrogen, and their nuclei were isolated. Immunoprecipitations were performed with the anti-Flag antibody and protein G beads. Immunoprecipitation in the absence of anti-Flag served as the control. qRT-PCR analysis was performed using specific primers corresponding to different promoter regions of *GmFT2a* and *GmFT5a*. *NbActin* was used as an internal control (primers used are shown in table S1).

### Electrophoretic mobility shift assay

For the *GmTOE4a*:*MBP* construct, the coding sequence of *GmTOE4a* was amplified and inserted into pMAL-c2X using Bam HI and Xba I. Recombinant GmTOE4a:MBP (maltose binding protein, MBP) protein was expressed in *E. coli* BL21 cells and purified using amyloseresin (New England Biolabs) according to the manufacturer’s instructions. The binding activity of the proteins was analyzed using an oligo nucleotide containing ACGACC motif present in the *GmFT2a* and *GmFT5a* promoters labeled with 6-FAM (6-carboxy fluorescein) at the 5′ end (Sangon Biotech, Shanghai, China). The annealing of the EMSA probe was performed as described above (*40*).

The reaction mixture (20 μl) contained EMSA/Gel-shift binding buffer (Biyuntian Institute of Biological Technology, China) and 5′ 6-FAM–labeled probe or unlabeled probe (unlabeled probes at 400× were used as specific competitors) and MBP or GmTOE4a:MBP protein. After incubation at room temperature for 25 min, gel electrophoresis was performed. Last, the typhoon 5 imager (GE General Corporation of America) was used at an emission of 517 nm and an excitation of 495 nm to see the results. The probe sequences used for EMSAs are listed in table S1.

### Statistical analysis

All data were analyzed using IBM SPSS Statistics 19 (IBM Corp., Armonk, NY). The means and SEs of all results were calculated, and analysis of variance (ANOVA) and Student’s *t* tests were performed to generate *P* values. Sigma Plot 10.0 (Systat Software Inc., San Jose, CA) and GraphPad Prism 9 (GraphPad Software) were used to generate graphs.

## Notes

View/request a protocol for this paper from *Bio-protocol*.
