# Supplementary material for: A nitrogen fixing symbiosis-specific pathway required for legume flowering
Source: Sci Adv. 2023 Jan 13;9(2):eade1150. doi: 10.1126/sciadv.ade1150 (PMC9839322; doi:10.1126/sciadv.ade1150)
Supplement: Supplementary file 1 — Figs. S1 to S14 Table S1 [file sciadv.ade1150_sm.pdf]

Science Advances
AAAS

Supplementary Materials for

A nitrogen fixing symbiosis-specific pathway required for legume flowering

Jinxia Yun *et al.*

Corresponding author: Wensheng Hou, houwensheng@caas.cn; Tianfu Han, hantianfu@caas.cn; Xia Li, xli@mail.hzau.edu.cn

Sci. Adv. **9**, eade1150 (2023)
DOI: 10.1126/sciadv.ade1150

This PDF file includes:

Figs. S1 to S14
Table S1

Fig. S1.

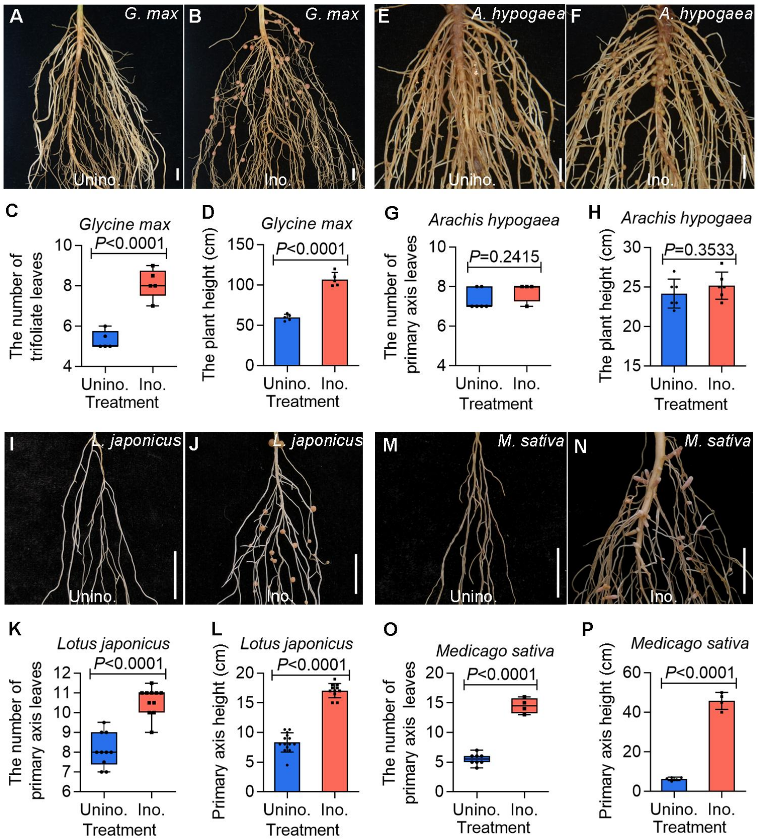

Fig. S1. Symbiotic nodulation and plant growth of legumes.

(**A** to **D**) The root from soybean (*Glycine max* cv. Jack) inoculated without (A) and with *Bradyrhizobium diazoefficiens* strain USDA110 (B) and the corresponding quantification data for leaves number (C) and plant height (D). (**E** to **H**) The root from peanut (*Arachis hypogaea* cv. Yuanza 6) without (E) and with *Bradyrhizobium yuanmingense* CCBAU 45321 (F) and the corresponding quantification data for leaves number (G) and plant height (H). (**I** to **L**) The root from birdsfoot trefoil (*Lotus japonicus* cv. MG-20) without (I) and with *Mesorhizobium loti* MAFF303099 (J) and the corresponding quantification data for leaves number (K) and plant height (L). (**M** to **P**) The root from alfalfa (*Medicago sativa* cv. Gannong 9) without (M) and with *Sinorhizobium meliloti* 2011 (N) and the corresponding quantification data for leaves number (O) and plant height (P). Bars = 1 cm, Data are means ± SDs ($n \geq 4$), and statistical significance was determined using Student's *t*-tests

Fig. S2.

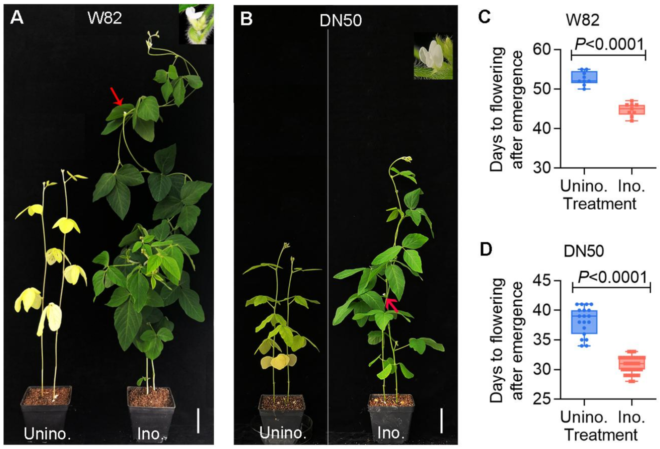

Fig. S2. Symbiotic nodulation has a similar accelerating effect on flowering in different soybean cultivars.

(**A** and **B**) Phenotype of soybean cultivars Williams 82 (W82) and Dongnong 50 (DN50) uninoculated (A) and inoculated with *Bradyrhizobium diazoefficiens* strain USDA110 (B), respectively. Red arrows indicate the first flowering buds. Photos were taken at the appearance of the first flowering. (**C** and **D**) Days from the emergence to flowering of the rhizobia uninoculated and inoculated W82 (C) and DN50 (D). Bars = 5 cm. Data are means ± SDs (n ≥ 10), and statistical significance was determined using Student's *t*-tests.

Fig. S3.

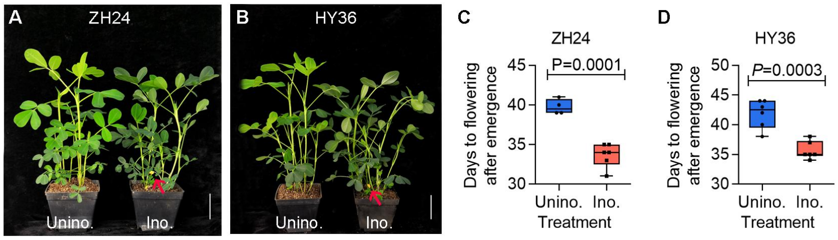

Fig. S3. Symbiotic nodulation accelerates flowering in different peanut cultivars.

(**A** and **B**) Phenotypes of peanut cultivars Zhonghua 24 (ZH24) (A) and Huayu 36 (HY36) (B) uninoculated and inoculated *Bradyrhizobium yuanmingense* CCBAU 45321, respectively. Red arrows indicate the first flowering buds. Photos were taken at the appearance of the first flowering. (**C** and **D**) Days from the emergence to flowering of the rhizobia uninoculated and inoculated peanut cultivars ZH24 (C) and HY36 (D). Bars = 5 cm, Data are means ± SDs (n ≥ 4), and statistical significance was determined using Student's *t*-tests.

Fig. S4.

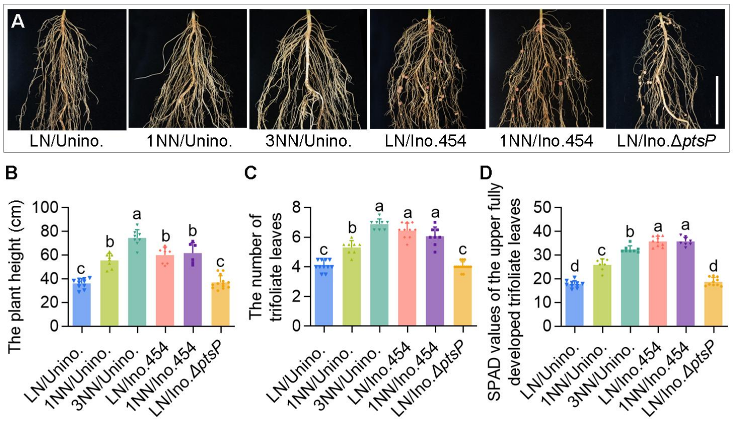

Fig. S4. Symbiotic nodulation and plant growth of uninoculated and inoculated DN50.

(**A** to **C**) The root phenotypes of DN50 uninoculated or inoculated with *S. fredii* CCBAU 45436 or the mutant *ΔptsP* strains under low nitrogen three times (LN) or low nitrogen twice with normal nitrogen once (1NN) or normal nitrogen three times (3NN) (A) and the corresponding quantification data for leaves number (B) and plant height (C). The symbols LN/Unino., 1NN/Unino., and 3NN/Unino. represent uninoculated plants grown under different nitrogen conditions; LN/Ino.454 and 1NN/Ino.454 represent plants inoculated with *S. fredii* CCBAU 45436 under LN and 1NN, and LN/Ino. *ΔptsP* means plants inculated with the mutant strain *ΔptsP* under low nitrogen condition. Bars = 5 cm. (**D**) SPAD values of the fully expanded youngest leaves of DN50 plant among different treatments. Data are means ± SDs (n ≥ 8), one-way ANOVA with Tukey's test was used for the statistical analysis ($P \leq 0.05$).

Fig. S5.

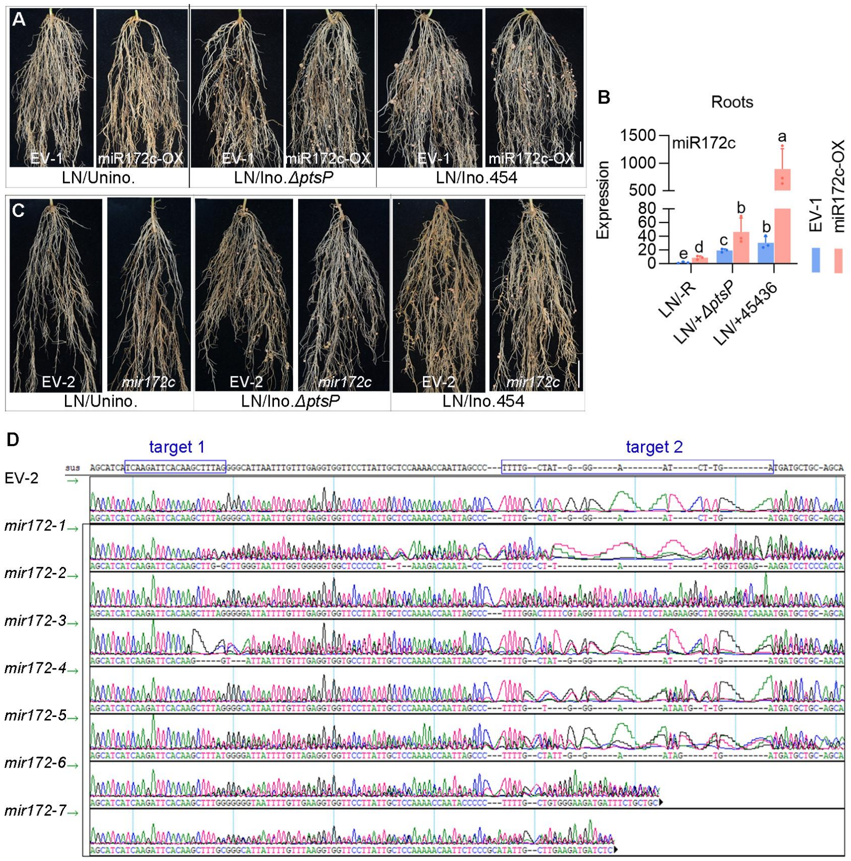

Fig. S5. Root phenotypes of miR172c overexpression and knockout composite plants, expression and editing types of miR172c.

(A) Root phenotypes of EV-1 and miR172c-OX uninoculated or inoculated with wild-type *S. fredii* CCBAU 45436- or *ΔptsP* mutant strains under low nitrogen condition. Bars = 2 cm. (**B**) qRT-PCR analysis of miR172c expression in above roots. Gene expression data are means ± SDs (n = 3). One-way ANOVA with Tukey's test was used for the statistical analysis ($P \leq 0.05$). (**C**) Root phenotypes of EV-2 and *mir172c* uninoculated or inoculated with wild-type *S. fredii* CCBAU 45436- and *ΔptsP* mutant strains under low nitrogen condition. Bars = 2 cm. LN/Unino.: plants had no rhizobial inoculation under LN; LN/Ino.454 and LN/Ino. *ΔptsP*: plants inoculated with *S. fredii* CCBAU 45436 or with the mutant strain *ΔptsP* under low nitrogen, respectively. (**D**) Sequencing results of miR172c gene editing in transgenic hairy roots. In EV-2 sequence shows two target sites and the underlined PAM motif.

Fig. S6.

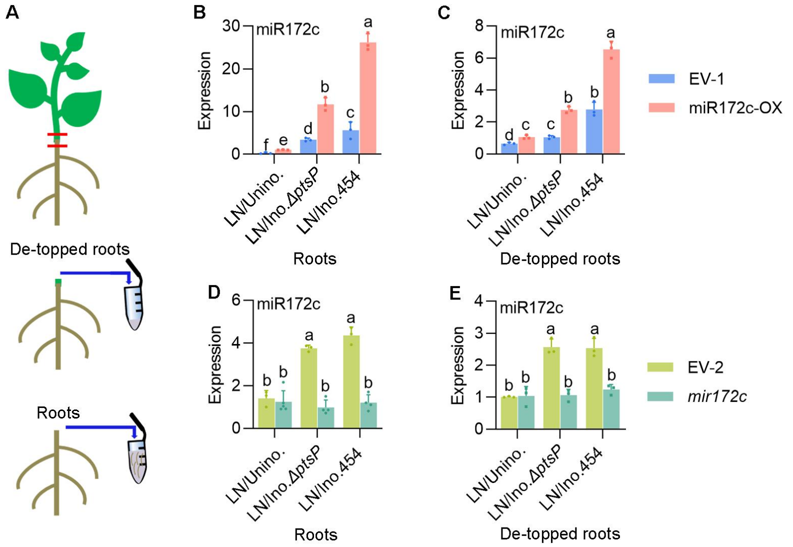

Fig. S6. The miR172c level in flow-out exudate.

(**A**) Schematic diagram of the location of phloem sap collection. De-topped roots represent cutting the aboveground part of the stem from 1-2 cm away from the root (the red line at the top), and collecting the phloem sap emerging from the roots. Roots (below the lower red line) represent cutting the root segment to collect its phloem sap. (**B** and **C**) qRT-PCR analysis of miR172c expression in flow-out excudate of the miR172c-OX plants and EV-1 (empty vector-1) plant de-topped roots (B) and roots (C). (**D** and **E**) qRT-PCR analysis of miR172c expression in flow-out excudate of the *mir172c* plants and EV-2 (empty vector-2) plant de-topped roots (D) and roots (E). Data are means ± SDs (n ≥ 3). One-way ANOVA with Tukey's test was used for the statistical analysis ($P \leq 0.05$).

Fig. S7.

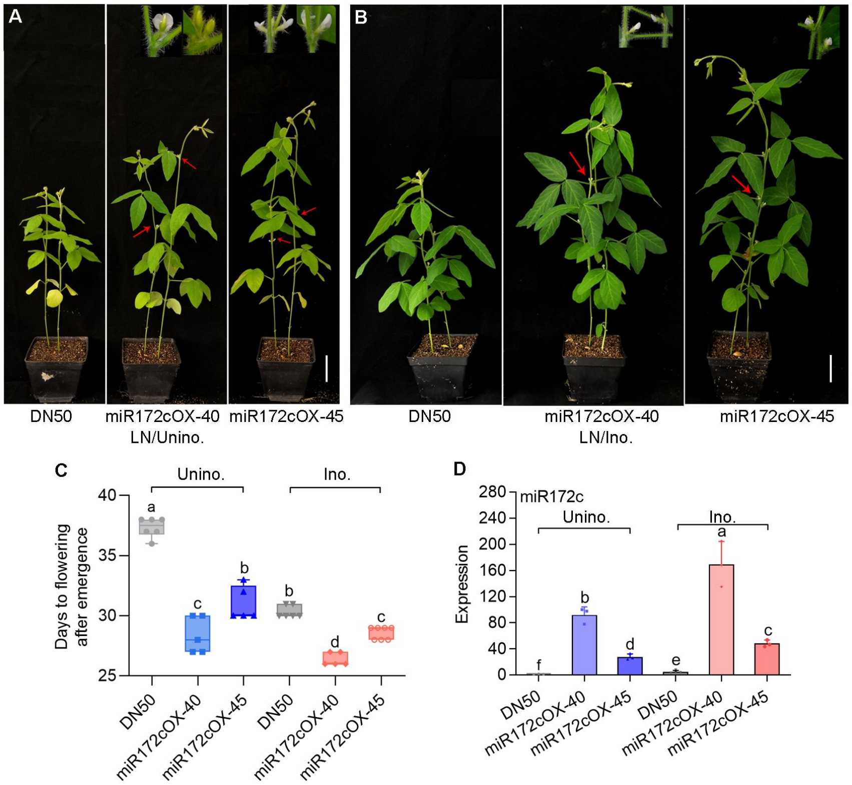

Fig. S7. Overexpression of miR172c causes early flowering of soybean.

(**A** and **B**) Phenotype of uninoculated (A) and inoculated *Bradyrhizobium diazoefficiens* strain USDA110 (B) DN50 and *35S::miR172c* transgenic lines (miR172cOX-40/45), respectively. Bars = 5 cm. The red arrows indicate the first flowering buds. (**C**) Days from emergence to flowering of rhizobia uninoculated and inoculated DN50 and miR172cOX-40/45. Data are means ± SDs (n ≥ 5). (**D**) qRT-PCR analysis of miR172c expression in leaves of DN50 and miR172cOX-40/45 rhizobia uninoculated and inoculated plants. Data are means ± SDs (n = 3). One-way ANOVA with Tukey's test was used for the statistical analysis ($P \leq 0.05$).

Fig. S8.

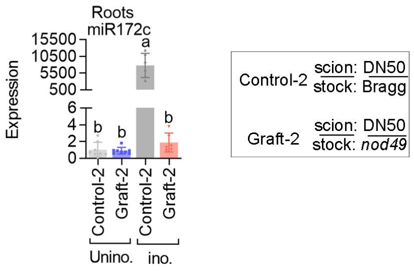

Fig. S8. qRT-PCR analysis of miR172c expression in roots of control-2 and graft-2 plants inoculated without or with rhizobia.

Bragg is a wild-type soybean cultivar, *nod49* is a loss of function mutant of Nod factor receptor GmNFR1α in Bragg background. Data are means ± SDs (n ≥ 5). One-way ANOVA with Tukey's test was used for the statistical analysis ($P \leq 0.05$).

Fig. S9.

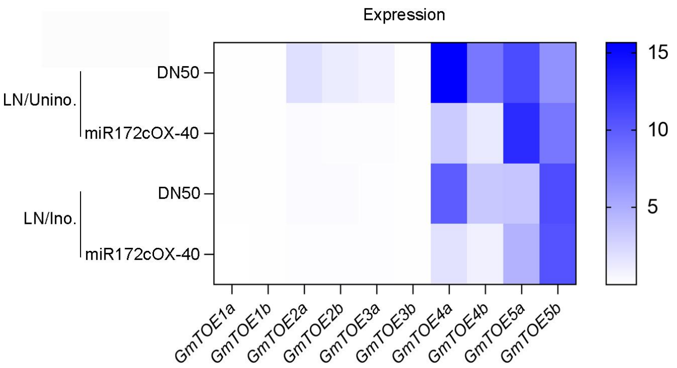

Fig. S9. Expression profile of *GmTOE* family members.

qRT-PCR analysis for the expression of *GmTOE* family members in leaves of DN50 and miR172cOX-40 transgenic plants uninoculated or inoculated with rhizobia *Bradyrhizobium diazoefficiens* strain USDA110 at 25 DAE. Data are means ± SDs (n ≥ 3).

Fig. S10.

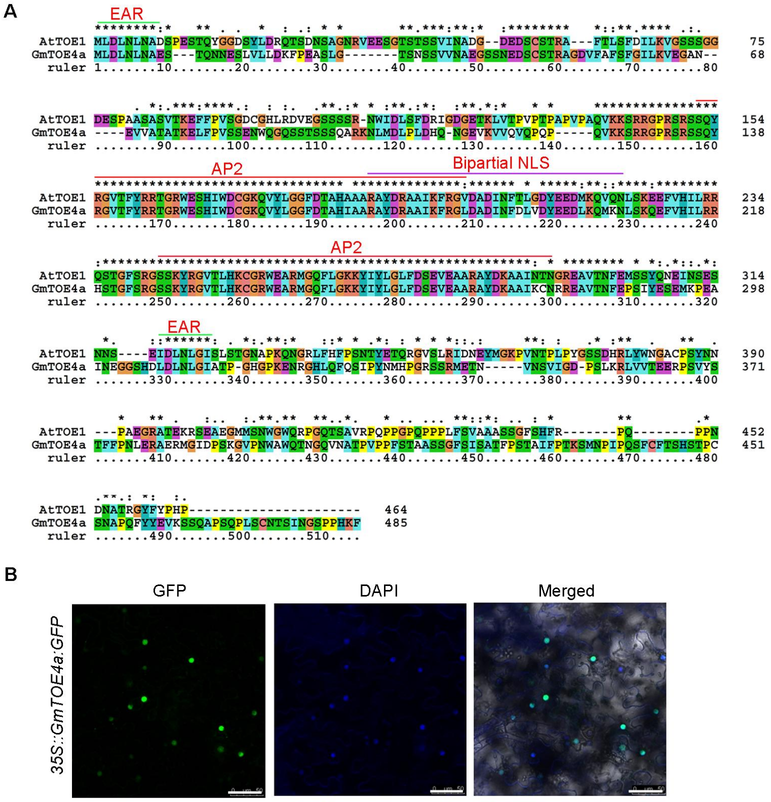

Fig. S10. GmTOE4a protein sequence and subcellular localization.

(**A**) The amino acid sequence alignment analysis between GmTOE4a and *Arabidopsis* AtTOE1. Both proteins have AP2 domains (red line indicates), the EAR motifs (green line indicates), and the bipartial NLS motif (purple line indicates). The alignment was analyzed using the software in https://rostlab.org/owiki/index.php/Predict. (**B**) GmTOE4a is located in nucleus of leaf cells in *Nicotiana benthamiana* and the nucleus was stained with DAPI (4', 6-diamidino-2-phenylindole). Bars = 50 μm.

Fig. S11.

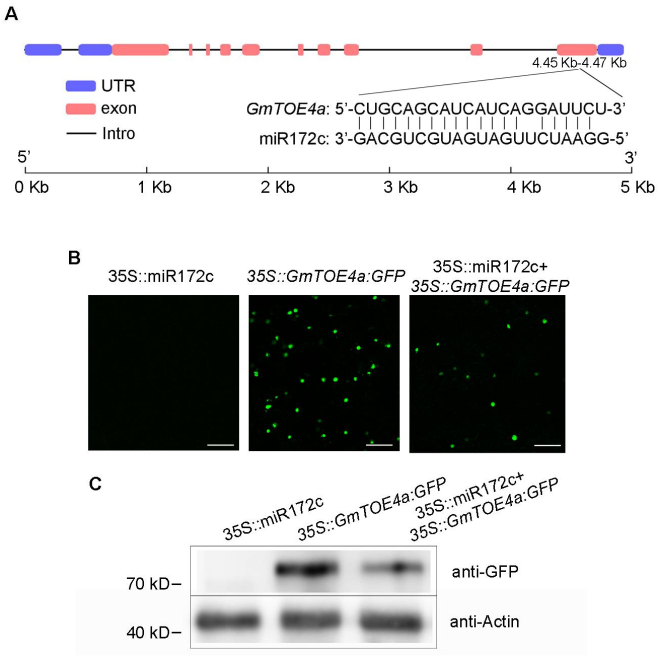

Fig. S11. *GmTOE4a* is the target of miR172c.

(**A**) *GmTOE4a* contains the miR172c target site in 10th exon. (**B**) Expression of *GmTOE4a* is suppressed by miR172c. 35S::miR172c and *35S::GmTOE4a:GFP* were transformed individually or cotransformed into *N. benthamiana* leaves. The images were taken 2 days after infiltration. The amounts of GmTOE4a:GFP fusion proteins were further detected by western blot with antibody against GFP, and the anti-actin was used as a loading control (**C**). Bars = 50 µm. Experiments were repeat three times with the same tendency.

Fig. S12.

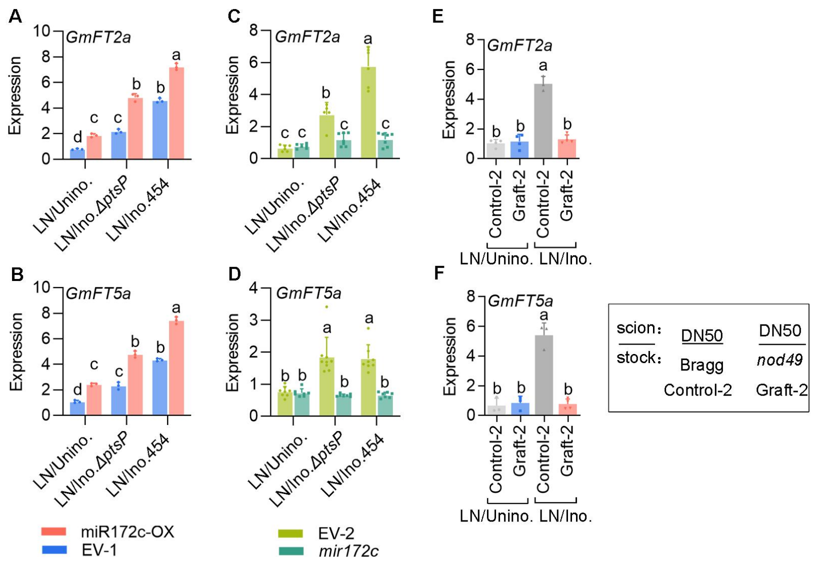

Fig. S12. Symbiosis-produced miR172c promotes flowering through activating *GmFT2a* and *GmFT5a*.

(**A** and **B**) qRT-PCR analysis the expression levels of *GmFT2a* and *GmFT5a* in leaves of EV-1 and miR172c-OX under different treatments. (**C** and **D**) qRT-PCR analyzing the expression levels of *GmFT2a* and *GmFT5a* in leaves of EV-2 and *mir172c* under different treatments. (**E** and **F**) qRT-PCR analysis of *GmFT2a* (E) and *GmFT5a* (F) expression in leaves of control-2 and graft-2 plants without or with rhizobia as shown in black box. Bragg is a wild-type soybean cultivar, and *nod49* is a loss of function mutant of Nod fact receptor GmNFR1α in Bragg background. Gene expression data are means ± SDs ($n \geq 3$). One-way ANOVA with Tukey's test was used for the statistical analysis ($P \leq 0.05$).

Fig. S13.

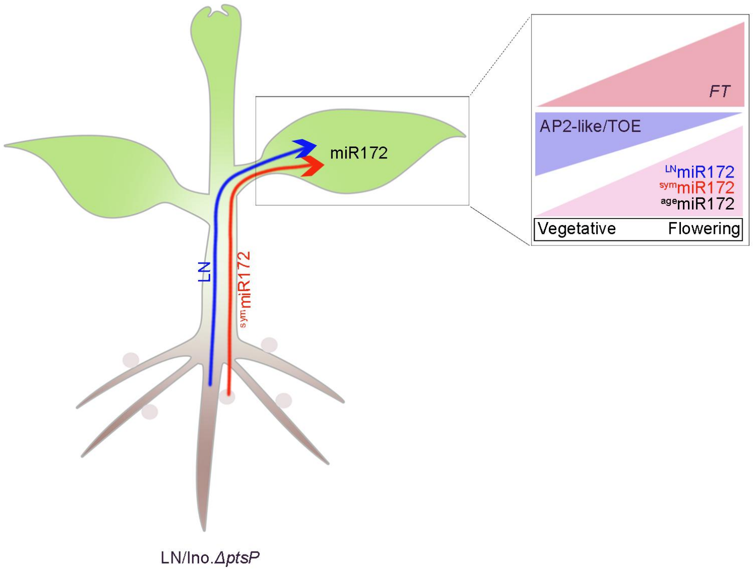

Fig. S13. Hypothetical mechanism of symbiotic signal-triggered early flowering.

Upon rhizobial inoculation with the mutant strain *ΔptsP*, the ineffective nodules produce miR172 (^{sym}miR172), which is transmitted from roots to leaves. In leaves, sum of the ^{sym}miR172s and age-related miR172 (^{age}miR172) in leaves causes an early peak in leaf miR172 abundance. miR172s reduce the abundance of the floral repressors AP2-like/TOEs and activate the flowering integrator *FTs*, thereby triggering early flowering. Blue line represents nitrogen nutrients from roots to leaves, red line indicates nodule-specific miR172 moving from roots to leaves.

Fig. S14.

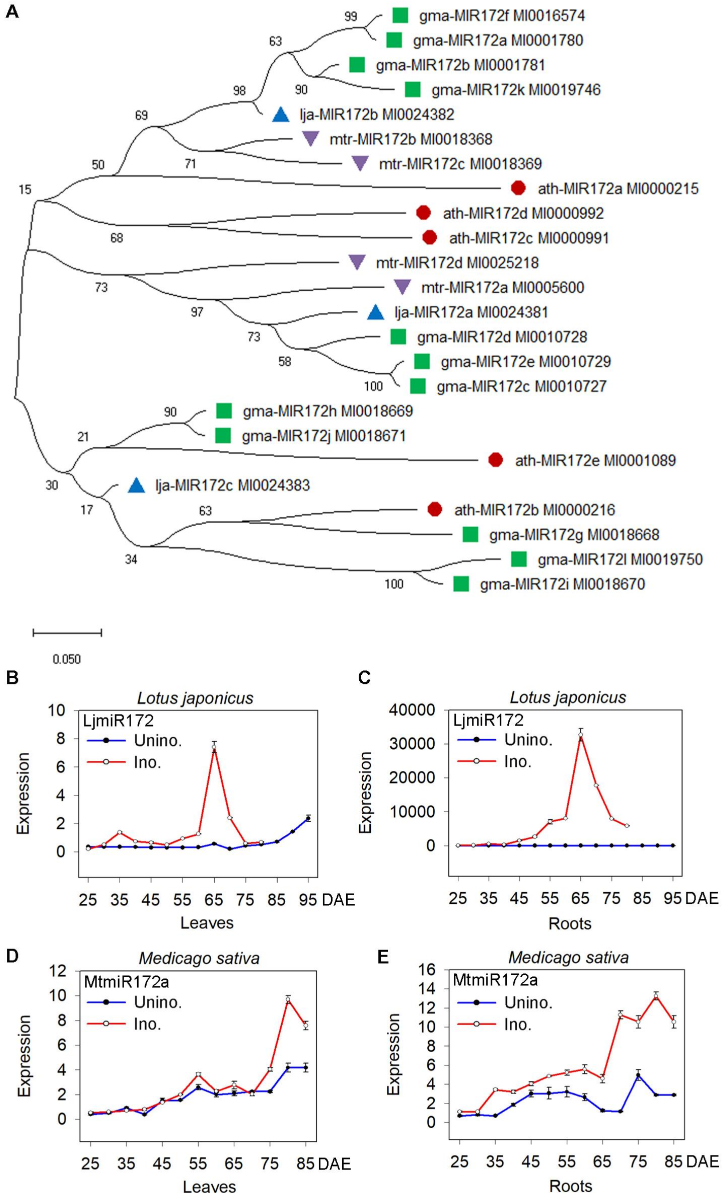

Fig. S14. The expression patterns of miR172 in legume plants inoculated with or without rhizobia.

(**A**) Phylogenetic tree for the miR172 family in Arabidopsis, birdsfoot trefoil, alfalfa and soybean. The tree was constructed using the maximum likelihood method based on the Tamura-Nei model. (**B** and **C**) The expression patterns of LjmiR172 in leaves (B) and roots (C) of rhizobia inoculated and uninoculated *L. japonicus* ecotype Miyakojima MG-20 at the specified time points. DAE, days after emergence. (**D** and **E**) The expression patterns of MtmiR172a in

leaves (D) and roots (E) of rhizobia inoculated and uninoculated *M. sativa* cultivar Gannong 9 at the specified time points. Gene expression data are means ± SDs ($n \geq 3$).

Table S1. Oligonucleotides used in this study.

Purpose	Gene	Orientation	Sequence (5'-3')
vector constructions	*MIR172c::GUS*	Forward	CGGGATCCCCCAATCCATAACAATACCC
		Reverse	CGGGATCCTTAGGACTTCATTAGGTCTCTCTG
	35S::miR172c	Forward	TGCACCGGTATGAAGTCCTAAATAAAC
		Reverse	CGGAATTCTCCTCCTCAAGCAATATCTG
	35S::TOE4a	Forward	GGGGACAAGTTTGTACAAAAAAGCAGGCTTCATGTTGGATCTTAATCTGAATGC
		Reverse	GGGGACCACTTTGTACAAGAAAGCTGGGTCGAACTTGTGTGGTGGGCTAC
	GmFT2a::GFP	Forward	GGGGACAAGTTTGTACAAAAAAGCAGGCTTCGGGTGAGAAGCACGAATAA
		Reverse	GGGGACCACTTTGTACAAGAAAGCTGGGTCGGGATAGTGTGCACACTAG
	GmFT5a::GFP	Forward	GGGGACAAGTTTGTACAAAAAAGCAGGCTTCCAATCAATCAGTGCCCAA
		Reverse	GGGGACCACTTTGTACAAGAAAGCTGGGTCGATTATTTACTTTAGATAG
		miR172c-DT1-BsF	ATATATGGTCTCGATTGCAAGATTCCCATAGCAAAAGTT

	premiR172c -pKSE401	miR172c-DT1-F0	TGCAAGATTCCCATAGCAAAAGTTTTAGAGCTAGAAATAGC
		miR172c-DT2-R0	AACCTAAAGCTTGTGAATCTTGCAATCTCTTAGTCGACTCTAC
		miR172c-DT2-BsR	ATTATTGGTCTCGAAACCTAAAGCTTGTGAATCTTGCAA
	GmTOE4a:MBP	Forward	CGGGATCCATGTTGGATCTTAATCTGAATGC
		Reverse	GCTCTAGAGAACTTGTGTGGTGGGCTAC
**reverse transcription and qPCR of miR172**	SL-gma-miR172c-RT (*40*)	GTCGTATCCAGTGCAGGGTCCGAGGTATTCGCACTGGATACGACCTGCAG	
	SL-miR1520d-RT (*40*)	GTCGTATCCAGTGCAGGGTCCGAGGTATTCGCACTGGATACGACTTGTCA	
	SL-Lj/Mt-miR172-RT (*47*)	GTCGTATCCAGTGCAGGGTCCGAGGTATTCGCACTGGATACGACCTGCAG	
	SL-U6-RT	GTGCAGGGTCCGAGGTTTGGACCATTTCTCGAT	
	gma-miR172c-qPCR	Forward	GGACCGGAATCTTGATGATG
	LjmiR172-qPCR (*47*)	Forward	GTGCGCAGAATCTTGATGATGCT
	MtmiR172a-qPCR	Forward	GTGCGCAGAATCCTGATGATGCT
	U6-qPCR	Forward	GGAACGATACAGAGAAGATTAGCA
	gma-miR1520d-qPCR	Forward	CGGACCATCAGAACATGACACG
	miRNA-universal	Reverse	GTGCAGGGTCCGAGGT

qRT-PCR	*GmFT2a* (*37*)	Forward	GGATTGCCAGTTGCTGCTGT
		Reverse	GAGTGTGGGAGATTGCCAAT
	GmFT5a-DB	Forward	TTATGCCAACTGCCAAAGAGAG
		Reverse	GGAGTAAGGCATCCAAGAATCT
	GmTOE1a-DB	Forward	CTTCAATTCCTCCGTCGTCAAT
		Reverse	GATCCACCTTCGACGAAAAATC
	GmTOE1b-DB	Forward	TGAATTTGCTTGGTTCTACAGC
		Reverse	TGGAGATACCAAAGTAAACGCT
	GmTOE2a	Forward	GCAAGTGGTTAGAGAGAAAG
		Reverse	GAGGTAGGTGTTGCAAAAATG
	GmTOE2b-DB	Forward	GTATTGGGCAGTTGTGGAATAC
		Reverse	CCAAGTTTGTGCTTTTGTTGTC
	GmTOE3a	Forward	AGAGATTGTTGAAGTTGAAGGTGGG
		Reverse	AGGAGTATTGGGAGAAGGAAGGAAG
	GmTOE3b	Forward	GTACGGTGATTATTTGATGAGAAGC
		Reverse	GTGAGAGGGAGAAAGAGAAAGAAGA
	GmTOE4a-DB	Forward	CACCACACAAGTTCTGAAGTTC
		Reverse	ACCATGAGCAACAACATTGTAC
	GmTOE4b-DB	Forward	GCTATCAAGTGCAATGGAAGAG
		Reverse	GCCACCTTCATTAATAGCTTCG
	GmTOE5a (NNC1) (40)	Forward	CAATGGGCAGGAAAGAGC
		Reverse	ATGGCAGTCGATGGAAAGGT
	GmTOE5b	Forward	GTGTTAGCGATACGGAAAACTC
		Reverse	GCTAAAGCCTGTTCCAACTAAC
	ELF1b	Forward	GTTGAAAAGCCAGGGGACA
		Reverse	TCTTACCCCTTGAGCGTGG

		Specific primers for these genes with -DB were obtained by directly querying the primer data qPrimerDB (https://biodb.swu.edu.cn/qprimerdb)	
**EMSA probes**	probe-*FT5a*	Forward	GTGTGGTGGTTCACGAGGGCACAAGGGTTC
		Reverse	GAACCCTTGTGCCCTCGTGAACCACCACAC
	probe-*FT2a*	Forward	TGTTTGAAAAAAACGAGGTGATCAGTTTTA
		Reverse	TAAAACTGATCACCTCGTTTTTTTCAAACA
**CHIP qPCR**	*FT2a*-ChIP-P1	Forward	GG CATAAATTTTGTAATTGGCCC
		Reverse	TTGCGTGCAT GTTCAGAGAC
	FT2a-ChIP-P2	Forward	GCGAATCCAAAGTGATGAAGA
		Reverse	TTCCCTTCTCGTCCTTTATAGC
	FT2a-ChIP-P3	Forward	G GGCACAATAAGTTTTTGGATC
		Reverse	ATGGAGGAGC AACGGAAGA
	FT2a-ChIP-P4	Forward	GAAAGACAAGGAGGAGCAATGG
		Reverse	GG ATAAAGAGAT CTTGACGGGT
	FT5a-ChIP-P1	Forward	GGTTATTGAGGATCATCACAGGC
		Reverse	ATTCGGTTTC ATACACCCAC G
	FT5a-ChIP-P2	Forward	GGCCTAAATCAGAAAATTGGTT
		Reverse	GGGACCTGAAGA GTAAACTCTG
	FT5a-ChIP-P3	Forward	GGGCTATGAAATGGAGATTTG
		Reverse	GCTCGTTACAA ATACCGTATT
	FT5a-ChIP-P4	Forward	GGGAGAGTGTGGTCCATATTAA
		Reverse	GG AGTAAACTCA ATGCATTGAG
	Nb-Actin	Forward	CTGAGAGATTCCGCTGC
		Reverse	GAGGACAATGTTTCCGTAC

**transgenic plant validation**	Bar	Forward	CTACATCGAGACAAGCACGGTCAA
		Reverse	AGAAACCCACGTCATGCCAGTTC
	miR172c-CrisperID	Forward	GATATGCTTCAATTCCTCCTCT
		Reverse	CTGGCCTTGTAGCACAGTTGT
